# Metabolite perturbations in type 1 diabetes associated with metabolic dysfunction-associated steatotic liver disease

**DOI:** 10.3389/fendo.2025.1500242

**Published:** 2025-06-03

**Authors:** Adeyinka Taiwo, Ronald A. Merrill, Linder Wendt, Daniel Pape, Himani Thakkar, J. Alan Maschek, James Cox, Scott A. Summers, Bhagirath Chaurasia, Nikitha Pothireddy, Bianca B. Carlson, Antonio Sanchez, Patrick Ten Eyck, Diana Jalal, Ayotunde Dokun, Eric B. Taylor, William I. Sivitz

**Affiliations:** ^1^ Division of Endocrinology, Department of Internal Medicine, University of Iowa Health Care, Iowa City, IA, United States; ^2^ Fraternal Order of Eagles Diabetes Center, University of Iowa, Iowa City, IA, United States; ^3^ Department of Molecular Physiology and Biophysics, University of Iowa Health Care, Iowa City, IA, United States; ^4^ Institute for Clinical and Translational Science, University of Iowa, Iowa City, IA, United States; ^5^ Roy J. and Lucille A. Carver College of Medicine, University of Iowa, Iowa City, IA, United States; ^6^ Department of Nutrition and Integrative Physiology, University of Utah, Salt Lake City, UT, United States; ^7^ Department of Biochemistry, University of Utah, Salt Lake City, UT, United States; ^8^ Department of Psychiatry and Behavioral Sciences, University of Washington, Seattle, WA, United States; ^9^ Division of Gastroenterology and Hepatology, Department of Internal Medicine, University of Iowa Health Care, Iowa City, IA, United States; ^10^ Center for Access and Delivery Research and Evaluation, Iowa City Veterans Affairs Health Care, Iowa City, IA, United States; ^11^ Renal Division, Department of Medicine, University of Iowa, Iowa City, IA, United States

**Keywords:** NAFLD, MASLD, NASH, MASH, Type 1 diabetes, Type 2 diabetes, FibroScan

## Abstract

**Background:**

Metabolic dysfunction-associated steatotic liver disease (MASLD), formerly called non-alcoholic fatty liver disease (NAFLD) is the hepatic manifestation of the metabolic syndrome. Although MASLD has been widely studied in persons with Type 2 diabetes (T2D), far less in known about the pathogenesis and severity of MASLD in Type 1 diabetes (T1D).

**Objectives:**

Determine metabolic perturbations associated with MASLD in persons with T1D.

**Study Design:**

We conducted a cross-sectional study of 30 participants with T1D. Based on the results of a FibroScan, participants were stratified as cases (MASLD) or controls. Metabolomic analyses were performed on plasma obtained from all participants after an overnight (after midnight) fast.

**Results:**

17 of 30 participants were classified as cases (MASLD) and 13 as controls. Cases had higher BMI (p=<0.001) and were taking higher daily insulin doses than controls (p=0.003). Metabolomic analyses revealed that those with MASLD had elevated levels of gluconeogenic substrates pyruvate (p=0.001) and lactate (p=0.043), gluconeogenic amino acids alanine (p<0.001) and glutamate (p=0.004), phenylalanine (p=0.003), and anthranilic acid (p=0.015). Lipidomics revealed, elevated ceramides (P=0.02), diacylglycerols (p=0.0009) and triacylglycerols (P=0.0004) in MASLD group. In those with MASLD, the acylcarnitines, isovalerylcarnitine (CAR.5.0) (P=0.002) and L-Palmitoylcarnitine (CAR.16.0) (P=0.048), were elevated. Pathway analyses using MetaboAnalyst 5.0 Software revealed that, pathways including phenylalanine and tyrosine metabolism, tryptophan metabolism, glucose-alanine cycle, glutamate metabolism, and glutathione metabolism were significantly enriched in those with MASLD.

**Conclusion:**

Participants with T1D and MASLD manifest features of insulin resistance and metabolite perturbations suggesting enhanced gluconeogenesis, dysfunctional fat synthesis, and perturbed TCA cycle activity.

## Introduction

Metabolic dysfunction-associated steatotic liver disease (MASLD), formerly called non-alcoholic fatty liver disease (NAFLD) is the most common form of chronic liver disease in the USA and developed countries with a rate in the adult population between 10-30% (1). MASLD consists of a spectrum of abnormalities with the earliest stage being simple steatosis, which progresses to metabolic dysfunction-associated steatohepatitis (MASH), formerly called non-alcoholic steatohepatitis (NASH). MASH consists of steatosis with hepatocyte ballooning, and inflammation with or without fibrosis and can further progress to cirrhosis, which is a risk factor for hepatocellular carcinoma (2). Cardiometabolic risk factors associated with the development of MASLD include obesity, insulin resistance and type 2 diabetes (T2D) (3). The global prevalence of MASLD among patients with T2D is estimated at 51-70% (3) and 22% in patients with Type 1 diabetes (T1D) (4). Younossi et al. estimated in 2017 that, the lifetime direct costs due to NASH in the United States would be $222.6 billion (5). The incidence of hepatic decompression, hepatocellular carcinoma, and death due to NASH cirrhosis are expected to increase 2 to 3-fold by 2030 (6).

The pathophysiology underlying MASLD is complex and incompletely understood. Hepatocyte fat accumulation is commonly associated with hyperinsulinemia and insulin resistance (7). The resultant lipotoxicity is believed to contribute to the severity of MASLD by inducing endoplasmic reticulum and oxidative stress, autophagy, apoptosis, and inflammation (7). Recent evidence indicates that obesity and insulin resistance in T1D are increasing over time (8), suggesting greater risk for MASLD. Although several studies have examined the pathogenesis of MASLD in T2D, studies of MASLD in T1D are limited and no study to date has evaluated the metabolomic profile associated with MASLD in TID. Studies of persons with T2D and obesity with MASLD showed that, increased levels of branched chain amino acids, aromatic amino acids, glutamate, alanine and ceramides, contribute to insulin resistance and hepatic dysfunction (9, 10).

To evaluate MASLD in T1D, we conducted a cross-sectional study. Participants with T1D were stratified as MASLD (cases) or controls, dependent on the results determined by FibroScan imaging. Key fasting intermediary metabolites, lipid species, and acylcarnitines were measured.

Given the lack of information regarding MASLD in type 1 diabetes and knowing that a substantial subset of individuals with type 1 diabetes are overweight or obese, we hypothesized that a random sample of participants with T1D would have a high prevalence of MASLD and those with MASLD would exhibit metabolites associated with insulin resistance including ceramides, glutamate, branched chain amino acids, and aromatic amino acids. We further hypothesize that these metabolites would correlate with the severity of MASLD as determined by FibroScan imaging.

## Research design and methods

### Study design

We conducted a cross-sectional study of 30 participants with T1D recruited from our university diabetes outpatient clinics. Inclusion criteria included: (i) age 18 and older and (ii) history of T1D with duration greater than 5 years. T1D was confirmed by evidence of either undetectable C-peptide or positive anti-glutamic acid decarboxylase antibody or typical history of T1D determined by an experienced diabetologist/endocrinologist in the setting of our diabetes specialty clinic. Exclusion criteria included: (i) alcohol consumption greater than 20 g/day in women or 30 g/day in men, (ii) hepatitis B or C infection, (iii) autoimmune liver disorders or other known metabolic causes of chronic liver disease such as hemochromatosis and Wilson’s disease, (iv) current pregnancy or lactation, (v) medications known to cause steatosis or (vi) illicit drug use. The study protocol was approved by our Institutional Review Board (approval #: 202405130). 250 individuals were invited to participate by letter or by face-to-face contact in our Endocrinology and Metabolism clinics. 35 responded positively. Of those 35, 30 met inclusion and exclusion criteria and were enrolled. Participants were recruited over a 7-month period from September 2021 to March 2022.

Participants were classified as cases (MASLD) if they had a controlled attenuation parameter (CAP) score (see below) of >=248 (mild or greater steatosis) or as controls if the CAP score was < 248 (11). Since there is variability in recommended CAP score cutoff points (12–14), we performed a sensitivity analysis using a CAP score of > 267 (moderate or greater steatosis) rather than >=248 for classification of cases versus controls. The specific data adjusted in this way appear in supplemental information.

Participants attended a single study visit in our Institutional Clinical Research Unit (CRU). Informed consent was obtained, blood was drawn for plasma metabolites and a liver FibroScan was performed. Demographic data including age, race, gender, and BMI was extracted from the history or electronic medical record. The total daily insulin dose was obtained from chart review and confirmed by the participant.

Participants were asked to avoid food intake from midnight until completion of their study visit. They were to take their scheduled long-acting insulin on the morning of their visit but omit their short acting pre-breakfast insulin dose. Participants were taking insulin either through a subcutaneous pump or receiving multiple doses including evening and/or bedtime insulin. As clinical goals include achieving fasting AM glucose control, participants entering our study had been treated to provide adequate basal insulin for this purpose. Therefore, we made no adjustments in insulin therapy on the night or day prior to study. If the pre-visit AM glucose was less than 70 mg/dl or greater than 200 mg/dl, participants were to reschedule their visit. At their study visit, 3 ml of blood was drawn into an EDTA containing tube for separation into plasma for subsequent metabolomic, lipidomic, and acylcarnitine analyses. Participants then had a FibroScan (Transient Elastography) done at our Gastroenterology Procedure Unit.

### Metabolite determinations

Fasting blood samples were placed on ice for 10 minutes, centrifuged, plasma extracted, and stored at -80C. All 30 plasma samples were processed and run together to prevent batch effects. Broad metabolite profiling was conducted using Gas Chromatography Mass Spectrometry (GC-MS). Lipidomics and acylcarnitines were measured using liquid chromatography tandem mass spectrometry (LC-MS). Comprehensive GC-MS and LC-MS methods are detailed in supplemental information. A total of 117 metabolites were measured by GC-MS and 14 acylcarnitines and 5 lipid species measured by LC-MS.

### FibroScan (liver elastography)

FibroScan was performed using an Echosense instrument (France) with either M or XL probes. The standard M probe was used when the skin-liver capsule distance (SCD) was less than 25 mm. The XL probe was used for patients with a larger SCD, including obese patients (BMI of 30 or above) and patients with a SCD of > 25 mm and a thoracic circumference of 75 cm or more, where the M probe can be unreliable due to interference from fat tissue. The FibroScan software automatically recommends the right probe size.

A liver stiffness score in kPa (kilopascal) was determined from measurement of shear wave propagation and used as a quantitative index of liver fibrosis stage, ranging from absent: Stage F0-1 (score < 8.2 kPa) through severe fibrosis Stage F4 (13.6 kPa) (11). A controlled attenuation parameter (CAP) score in dB/m (decibels per meter) was determined and used as a quantitative index of hepatic steatosis ranging from absent (S0: < 248), mild steatosis (S1: 248-267), moderate steatosis (S2: 268-279) to severe steatosis (S3: > 280) (11). We used the FibroScan to diagnose MASLD as opposed to the gold standard liver biopsy, due to the invasive nature and possible morbidity associated with biopsies.

### Statistical analyses

After metabolite peak areas and the NOREVA correction were performed as described in the supplementary methods, values for individual metabolites were normalized by per sample total metabolite signal. Per sample-normalized individual metabolite values were then divided by the mean of the control group for that metabolite, leading to individual metabolites being reported on a fold-of-control basis. Categorical variables were summarized using counts and percentages, while continuous demographic variables were summarized using either means and standard deviations or medians and inter-quartile ranges. The choice between the two methods depended on the normality of the distribution as determined by a Shapiro-Wilk test. Continuous variables with a Shapiro-Wilk p-value less than 0.05 were considered non-normally distributed, while the remaining continuous variables were considered to be approximately normal.

To assess differences in demographic variables between cases and controls, Fisher’s Exact Test was utilized for categorical variables. For the non-normal and approximately normal continuous variables, the Wilcoxon rank sum test and Welch two sample t-test were employed respectively. In addition, correlations between continuous measures of interest were also evaluated using Spearman’s correlation.

To construct regression models to predict fibrosis and steatosis scores, a stepwise selection algorithm was used to find the 3-predictor model for each outcome variable that has the lowest Akaike Information Criterion (AIC). All metabolites and acylcarnitines were considered as candidates for inclusion into the model, as well as BMI and 24-hour insulin dose. This algorithm began by fitting an intercept-only model and then examining whether the addition of independent variables would improve the AIC of the model. This process was continued until the model had 3 independent variables, and then the algorithm was terminated. A linear regression model was used to predict steatosis scores due to their approximately normal distribution, while a log-transformed Gamma model was used to predict fibrosis scores due to the right-skewed nature of fibrosis scores in our study. Point and interval estimates were calculated for predictors in each optimal model, along with p-values.

P-values less than 0.05 were considered statistically significant. False Discovery Rate-adjusted p-values were calculated using the Benjamini & Hochberg correction and are presented to provide context for interpretation of the data given the many comparisons that were performed. Data analyses were carried out using R version 4.2.3, GraphPad Prism version 9, and by the MetaboAnalyst 5.0 software package.

## Results

### Demographics

Participants characteristics are listed in **Table 1**. Of 30 participants, 13 were controls and 17 were cases. Those with MASLD versus the controls were of comparable age and gender. The group with MASLD had a significantly higher BMI (p <0.001) and were taking higher daily insulin doses (p=0.003). There were no statistically significant differences in age, race, gender or hemoglobin A1C. The mean duration of diabetes was significantly longer in the controls than those with MASLD. Sensitivity analyses using a CAP score of ≤ 267, which encompassed 16 controls and 14 cases, showed that the duration of diabetes lost significance but was still longer in the case cohort (p =0.08). Moreover, not surprisingly the difference in fibrosis staging became significant (more controls in mild stage) when classified by CAP score ≤ 267.

**Table 1:** Participant demographics.

Characteristic	CONTROL, N = 13^1^	CASE, N = 17^1^	p-value^2^
AGE (yrs)	38 (34, 42) [Min: 25, Max: 67]	41 (29, 65) [Min: 23, Max: 72]	0.66
SEX			
Female	7 (54%)	8 (47%)	
Male	6 (46%)	9 (53%)	
Duration of DM (yrs)	25 (17, 39) [Min: 10, Max: 61]	13 (13, 17) [Min: 3, Max: 61]	**0.032**
Mean HbA1C (%)	7.10 (1.31)	7.58 (1.12)	0.299
BMI (kg/m² )	28 (5)	37 (7)	**< 0.001**
24hrs Insulin dose (units)	44 (35, 54)	67 (57, 115)	**0.003**
Insulin unit per Kg	0.51 (0.47, 0.70) [Min: 0.34, Max: 1.35]	0.69 (0.59, 0.91) [Min: 0.38, Max: 2.61]	0.062
RACE			0.433
Black	1 (7.7%)	0 (0%)	
Caucasian	12 (92%)	17 (100%)	

^1^Median (IQR); n (%); Mean (SD).

^2^Wilcoxon rank sum test; Fisher's exact test; Welch Two Sample t-test.

The bold values are statistically significant with p <0.05.

### Steatosis and fibrosis

FibroScan results (**Table 2**) showed that cases had a higher mean steatosis score than controls. All cases had steatosis, with 13 out of 17 (76%) having moderate to severe steatosis (Stage S2-S3). Four out of 17 (24%) in the case group had evidence of fibrosis of stage F2 or greater. A comprehensive list of our FibroScan results is shown in **Supplementary Table 1**.

**Table 2:** FibroScan results.

Characteristic	Controls, N = 13^1^	Cases, N = 17^1^	p-value^2^
**Steatosis Score**	202 (20)	312 (44)	**< 0.001**
**Steatosis Stage**			**< 0.001**
S0	13 (100%)	0 (0%)	
S1	0 (0%)	4 (24%)	
S2	0 (0%)	3 (18%)	
S3	0 (0%)	10 (59%)	
**Fibrosis score**	4.5 (1.2)	7.4 (3.6)	**0.005**
**Fibrosis Stage**			0.492
F0-F1	13 (100%)	13 (76%)	
F2	0 (0%)	1 (5.9%)	
F3	0 (0%)	2 (12%)	
F4	0 (0%)	1 (5.9%)	

^1^Mean (SD); n (%).

^2^Welch Two Sample t-test; Fisher's exact test.

The bold values are statistically significant with p <0.05.

**Figure 1:**
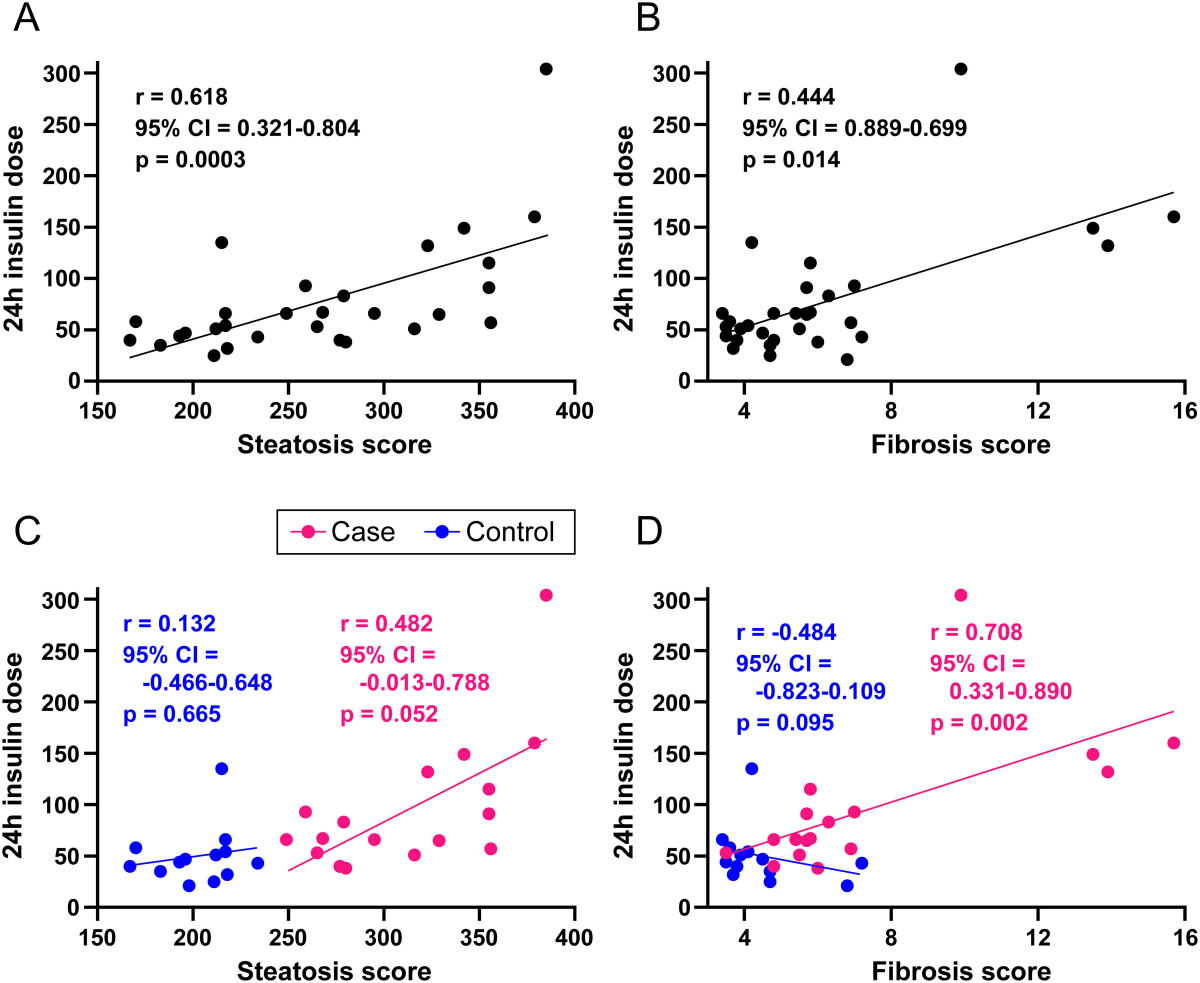
Correlations between 24h insulin dosage with steatosis and fibrosis scores. Data are shown separately for combined cases and controls **(A-B)** and individually for cases and controls **(C-D)**. r values by Spearman correlation. Lines by linear regression.

*Association between 24h insulin dosage and severity of steatosis or fibrosis:* Among all participants, we observed a positive correlation between daily insulin dosing with both steatosis and fibrosis scores (**Figures 1A, B**). Upon stratification, the correlation among the case group for fibrosis score with daily insulin dosing remained significant while steatosis correlated at a p value of 0.052 (**Figures 1C, D**). Sensitivity analysis revealed that the correlation of steatosis with insulin dosing among the case group became significant at a p value of 0.025.

### Metabolite determinations

**Figure 2:**
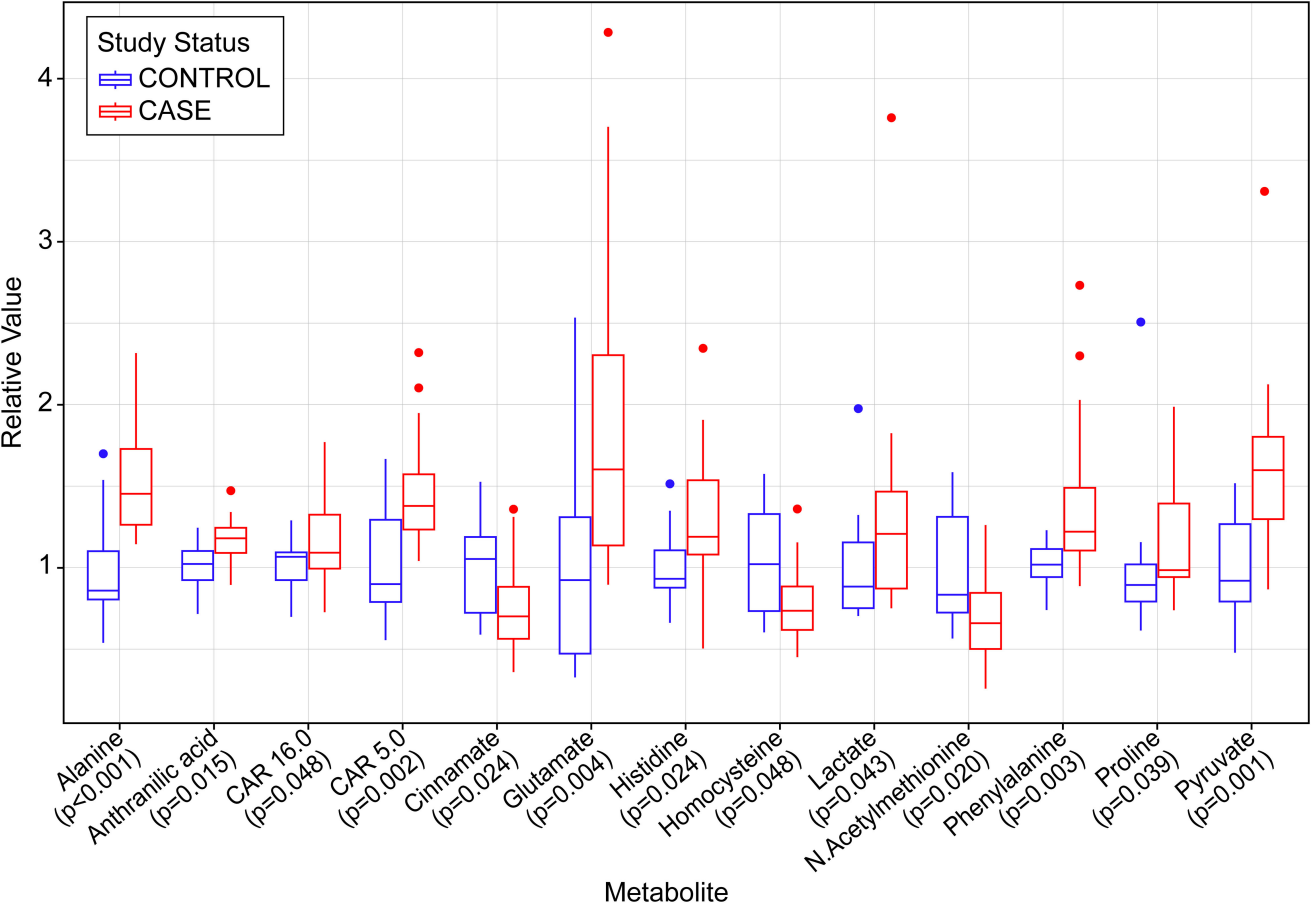
Boxplot displaying relative levels of metabolites that differ significantly between cases (red) and controls (blue, control values normalized to 1.00). Data are displayed via medians and interquartile ranges, with outliers denoted by points on the graph. P-values were calculated via the Welch two sample t-test for approximately normal distributed data according to the Shapiro-Wilk test or by the Wilcoxon rank sum test for data that were not normally distributed.

117 metabolites were assessed by GC-MS. **Figure 2**; **Supplementary Table 2** show that the aromatic amino acid, phenylalanine, was higher in the group with MASLD versus controls (p=0.003), but the other aromatic amino acids, tryptophan and tyrosine, did not differ significantly among groups. Dihydroxyphenylalanine, a metabolite of tyrosine was higher in cases and trended towards significance at p=0.053. Isoleucine, a branched chain amino acid was higher in cases and trended towards significance at p=0.05. Anthranilic acid, a metabolite of tryptophan, was significantly elevated in MASLD (p=0.015). Other metabolites elevated in the MASLD included glutamate (p=0.004), histidine (p=0.024), proline (p=0.039), and the major gluconeogenic substrates; lactate (p=0.043), pyruvate (p= 0.001), and alanine (p=<0.001). Metabolites significantly reduced in the MASLD group included cinnamate (p=0.024), homocysteine (p=0.048) and N-acetyl-methionine (p=0.020). We also quantified 14 species of plasma acylcarnitines using LC-MS. Among these CAR 5.0 (p = 0.002) and CAR 16.0 (p = 0.048) were significantly greater in the MASLD group: **Supplementary Table 3**. A comprehensive list of all measured metabolites and acylcarnitines and are shown in **Supplementary Tables 2, 3**, respectively. Using the CAP of ≤ 267, sensitivity analyses revealed loss of significance for anthranilic acid, histidine, proline, cinnamate, and homocysteine. The gluconeogenic metabolites; lactate, pyruvate, and alanine remained significantly elevated in the case group. The acylcarnitine, CAR 5.0 remained elevated in the case group but CAR 16.0 lost significance.

### Metabolite associations with BMI, steatosis and 24 h insulin dosage

**Figure 3:**
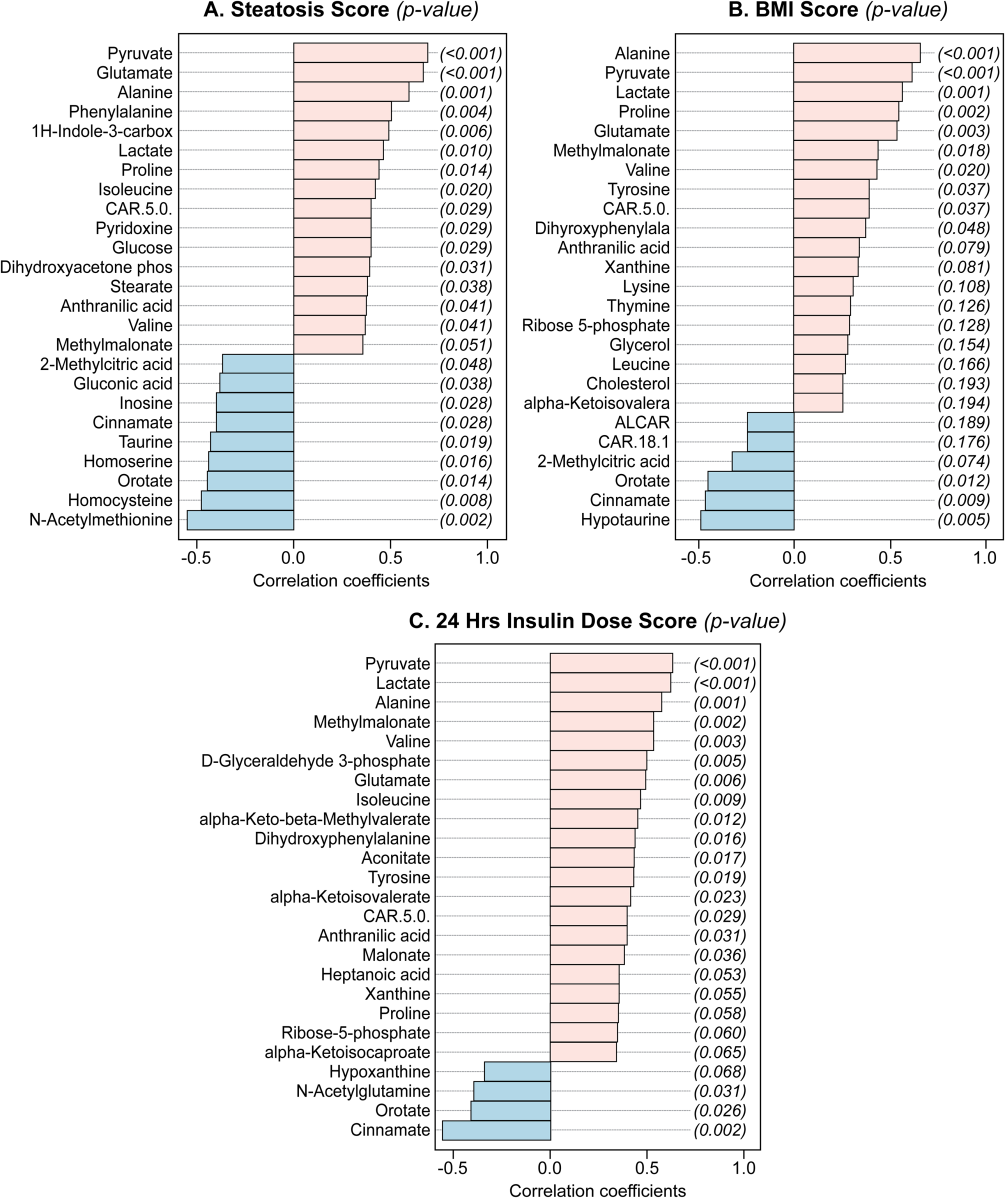
Top 25 metabolites as correlated to steatosis **(A)**, BMI **(B)**, and 24 h insulin dosing **(C)**.

**Figure 3** depicts the top 25 metabolites that correlated with steatosis (**Figure 3A**), BMI (**Figure 3B**), or total daily insulin dosage (**Figure 3C**). Glutamate, pyruvate, alanine, valine, lactate, and CAR 5.0 correlated positively and significantly with BMI, steatosis, and 24h insulin dosage with variable results for other compounds. Orotate, and cinnamate correlated negatively and significantly with BMI, steatosis and 24h insulin dosage. Of the aromatic amino acids, only phenylalanine correlated positively with the steatosis score. Tyrosine correlated positively with BMI and 24h insulin dosage. Dihydoxyphenylalanine, a metabolite of tyrosine, also correlated positively with BMI and 24h insulin dosage. Proline correlated positively with BMI and steatosis and had a near significant positive correlation with 24h insulin dosage (p=0.058). Methylmalonate correlated positively with BMI and 24h insulin dosage and had a near significant positive correlation with steatosis (p=0.051).

### Magnitude and fold change for individual metabolites

**Figure 4:**
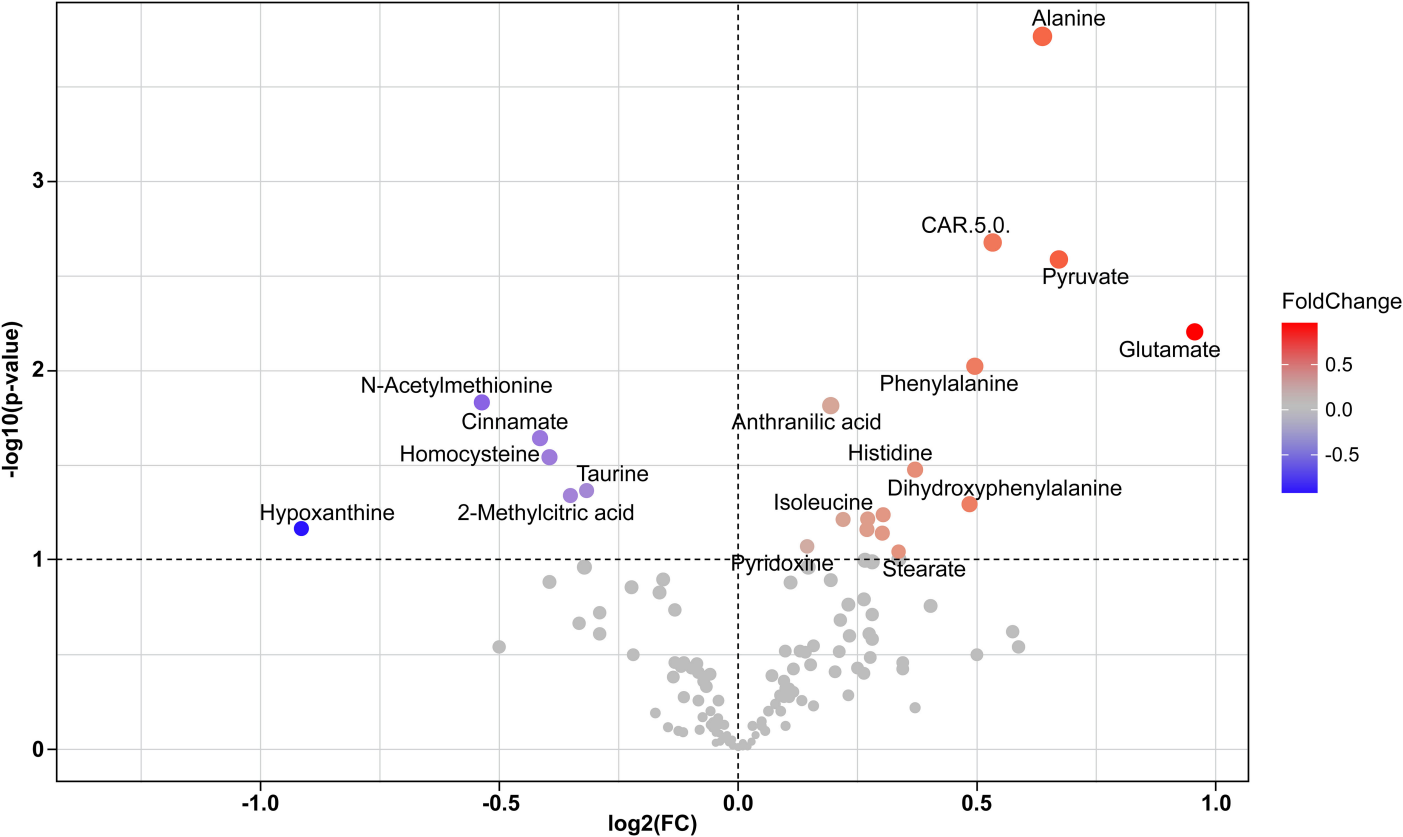
Compound levels depicted by Volcano plot. Upward and right direction favor greater levels in cases compared to controls. 117 metabolites and 14 acyl carnitines measured by LCMS were entered and analyzed using the MetaboAnalyst 5.0 software package.

**Figure 4** shows a Volcano plot determined by entry of 117 measured metabolites and 14 acyl carnitines into MetaboAnalyst 5.0 software. Alanine, pyruvate, glutamate, Car 5.0, and phenylalanine levels were notably higher in cases compared to controls with substantial positive fold change. Sensitivity analysis with a CAP cutoff ≤ 267 revealed the same directional and similar magnitude of these findings.

### Pathway enrichment

**Figure 5:**
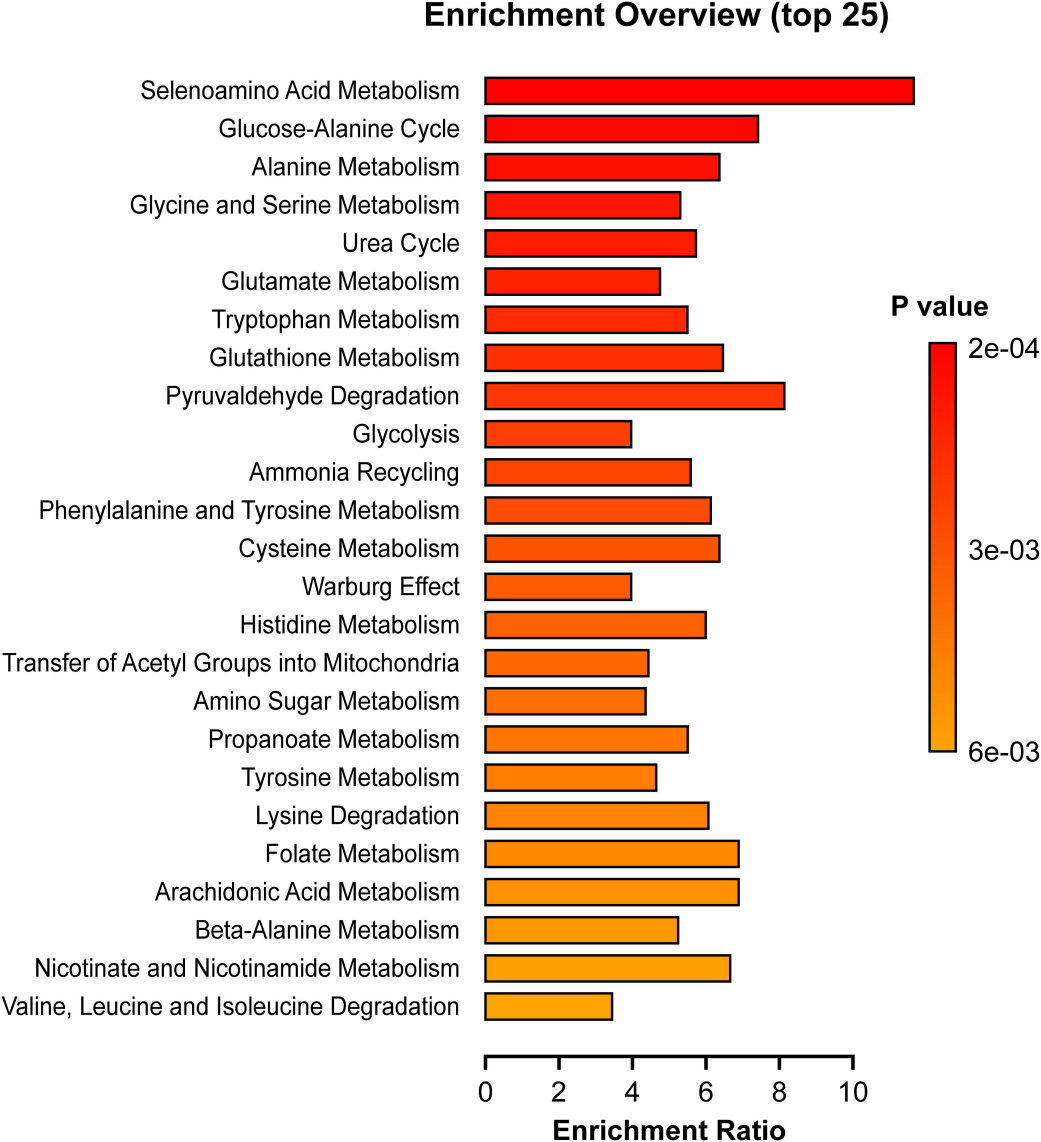
Pathway Enrichment Analyses. Depicted pathways differed significantly between cases and controls (p < 0.05). The enrichment ratio was determined using MetaboAnalyst 5.0 and calculated as the number of hits within a particular metabolic pathway divided by the expected number by chance.

We investigated whether certain functionally related metabolites were significantly enriched through hits compared with a database of 99 metabolic sets available in the library of the MetaboAnalyst 5.0 software (**Figure 5**). Of particular interest, the glucose-alanine cycle, phenylalanine and tyrosine metabolism, glutathione metabolism, alanine metabolism, tryptophan metabolism, cysteine metabolism and glutamate metabolism were significantly enriched in cases (p <0.05). Sensitivity analysis with a CAP cutoff ≤ 267 revealed the same findings with similar magnitude.

### Lipidomics

Limited lipidomic analyses were done using LC-MS. The results (**Table 3**) showed that ceramides (p = 0.02), diacylglycerols levels (p = 0.0009), and triacylglycerols (p=0.0004) were significantly higher in the MASLD group. Our metabolite profile included the non-esterified fatty acids; stearate, palmitate, laurate, and linoleate (**Supplementary Table 2**). Stearate was mildly elevated in cases versus controls (mean ± SD, 1.26 ± 0.44 *vs*. 1.00 ± 0.35) but missed significance at p = 0.08 while the others were very similar between groups. Sensitivity analyses showed that the data in **Table 3** and the non-esterified fatty acid data was essentially not affected.

**Table 3:** Lipid classes determined by LC-MS.

Lipid species (nmol/ml)	Control [Median (interquartile range)] (n = 13)	Cases [Median (interquartile range)] (n = 17)	p-value	FDR p-value
Ceramides	4.54 (3.89 – 4.87)	5.40 (4.57 – 6.81)	**0.02**	**0.033**
Sphingomyelin	374.90 (315.30 – 403.90)	372.90 (325.20 – 440.60)	0.48	0.48
Diacylglycerols	33.16 (29.16 – 44.16)	51.20 (43.58 – 83.38)	**0.0009**	**0.0023**
Phosphatidylcholine	1184.0 (1125.0 – 1324.0)	1275.0 (1190.0 – 1548.0)	0.13	0.16
Triacylglycerols	531.70 (380.70 – 658.90)	789.60 (623.60 – 1695.0)	**0.0004**	**0.0020**

Differences between groups were determined using a Wilcoxon rank sum test. Data show median and interquartile range with significant differences indicated in bold type.

### Multivariate regression model

Results from **Table 4** show that elevated glutamate and phenylalanine levels predicted a greater steatosis score, while higher aspartate levels predicted a lower steatosis score. **Table 5** shows that increased BMI was associated with higher fibrosis scores, while asparagine and sedoheptulose were associated with lower fibrosis scores.

**Table 4:** Multivariate modeling to predict steatosis.

Characteristic	Beta	95% CI^1^	p-value
Glutamate	67	48, 86	**<0.001**
Phenylalanine	72	42, 102	**<0.001**
Aspartate	-104	-162, -46	**0.001**

^1^CI = Confidence Interval.

When glutamate values increase by 1, we anticipate that the steatosis score will increase by 67 units. When phenylalanine values increase by 1, we anticipate that the steatosis score will increase by 72 units. When aspartate values increase by 1, we anticipate that the steatosis score will decrease by 104 units. p=statistical significance = <0.05.

The bold values are statistically significant with p <0.05.

**Table 5:** Multivariate modeling to predict fibrosis.

Characteristic	exp(Beta)	95% CI^1^	p-value
BMI, 5 Unit Increase	1.19	1.12, 1.26	**<0.001**
Asparagine	0.57	0.41, 0.80	**0.005**
Sedoheptulose	0.58	0.35, 0.95	**0.039**

^1^CI = Confidence Interval.

When BMI increases by 5, we anticipate a multiplicative change in their fibrosis score by 1.19. When asparagine increases by 1 unit, we anticipate a multiplicative change in the fibrosis score by 0.57. When sedoheptulose values increase by 0.58, we anticipate a multiplicative change in their fibrosis score by 0.58.

The bold values are statistically significant with p <0.05.

## Discussion

To our knowledge, this is the first study of MASLD in persons with T1D employing detailed metabolomic analyses. We found that over half of our participants had MASLD. Those with MASLD manifest features of insulin resistance including higher mean BMI and greater insulin dose requirements. Moreover, as discussed below our metabolite data was consistent with values associated with insulin resistance including elevated aromatic amino acids, metabolites involved in gluconeogenesis, acyl carnitines, ceramides, diacylglycerols and triacyclglycerols.

As compared to T2D, much less is known regarding the pathogenesis of MASLD in T1D. This is likely because of the presumed lower incidence of MASLD in T1D and exclusion of persons with T1D from MASLD studies. Over half (57%) of the recruited T1D participants in our study were found to have MASLD, with 24% in this case group having evidence of fibrosis (stage F2 or greater, i.e., fibrosis score 8.2 or greater). This is notable and suggests that the prevalence of MASLD in T1D, as we defined by steatosis on FibroScan, may be higher than reported in a meta-analysis that used a variety of criteria for the diagnosis of MASLD in persons with T1D (4). Long-term studies of patients with MAFLD have confirmed that hepatic fibrosis stage F2 or higher is associated independently with liver-related mortality (15, 16).

Due to the complications of MASLD including cardiovascular and liver morbidity, mortality, and economic burden, it is important to better understand the pathogenesis of MASLD in T1D. Overall, we observed several similarities between the metabolic profile of the MASLD group in T1D and what is historically well recognized in persons with T2D. Notably our case group with MASLD exhibited significantly higher levels of alanine, proline, histidine, isoleucine, phenylalanine, glutamate, acyl carnitines (Car 5.0 and Car 16.0), ceramides, diacylglycerols and triacylglycerols, which are findings reminiscent of T2D with MASLD (9, 10, 17, 18). The MASLD group had higher levels of glucose, alanine, pyruvate, and glutamate compared to controls. These compounds represent key metabolites involved in the glucose-alanine cycle (Cahill Cycle), a pathway active in the presence of hepatic insulin resistance and found enriched in our pathway data (**Figure 5**). In this process, muscle glutamate reacts with pyruvate generating alanine and α-ketoglutarate catalyzed by alanine aminotransferase. Alanine can then be used by liver for gluconeogenesis (19, 20). Lactate was also greater in cases, which along with the elevated pyruvate, imply greater activity of the Cori Cycle. In this cycle, anaerobic glycolysis in muscle directs lactate to liver for reconversion to pyruvate used for glucose production contributing to the gluconeogenesis associated with MASLD. Also, phenylalanine and tyrosine metabolism were enriched in cases compared to controls consistent with reports that aromatic amino acids are increased with insulin resistance and obesity (21, 22). Moreover, we observed that the group with MASLD had higher levels of anthranilic acid, a metabolite of tryptophan, whose breakdown products are associated with obesity and insulin resistance (22).

Plasma levels of branched chain amino acids (BCAAs) have been shown to be elevated in obesity associated MASLD (9) due to impaired BCAA metabolism in the setting of mitochondrial dysfunction and hepatic inflammation (23). In this regard, it was suggested that elevated BCAAs may represent an adaptive response to inflammation and oxidative stress (10). In any case, there is a strong association of BCAAs with features of type 2 diabetes including insulin resistance, diabetes, and cardiovascular risk (24). Somewhat in contrast, our study of individuals with T1D revealed only a marginally significant increase in isoleucine: **Supplementary Table 2**, with non-significant increases in leucine and valine in cases versus controls: **Supplementary Table 2**.

We also found higher circulating levels of glutamate (**Figures 2**, **4**) and enriched glutamate metabolism (**Figure 5**) in cases versus controls. We also observed that glutamate emerged as a predictor of steatosis in multivariate modeling (**Table 4**). However, we observed no difference in plasma glutamine, the dietary precursor of glutamate after mitochondrial conversion by glutaminase. There is evidence that glutamine supplementation may be preventative towards liver fat accumulation and the metabolic risk for cardiovascular disease (25). On the other hand, there is evidence that plasma glutamate may be a marker of liver fat accumulation, fibrosis, and cardiovascular risk (26, 27). Our current observation of higher glutamate in cases is consistent with this. Although glutamate is important in glutathione synthesis, a major antioxidant mechanism preventing hepatic inflammation and fibrosis (28), this may be a compensatory response (29). Finally, there is evidence that a higher glutamine-to-glutamate ratio is associated with decreased cardiometabolic risk while a higher glutamate concentration is associated with increased risk (30).

In our study the case group had a near significant (p=0.053) lower level of taurine compared with the controls: **Supplementary Table 2** and taurine correlated negatively with steatosis (**Figure 3a**). Taurine is synthesized in the liver from cysteine and methinione metabolism (31) and it ameliorates hepatic steatosis by reducing reactive oxygen species, lipid accumulation, and preserving mitochondrial membrane potential (32).

The quantity and quality of accumulated lipids play a significant role in the pathogenesis of MASLD (33). In this regard, we observed that diacylglycerols, triacylglycerols and ceramides were significantly higher in the case group compared to controls. Prior lipidomic studies have shown sphingomyelin, ceramides, and dihydrate ceramides in plasma and liver biopsies of patients with MASLD and MASH (34). Ceramides are sphingolipids that accumulate in the liver and are involved in increased oxidative stress, mitochondrial dysfunction and cell apoptosis (33). Hepatic ceramides accumulation is believed to be involved in the pathogenesis of MASLD by interfering with signaling through disruption of the insulin receptor substrate and protein kinase B (35). Diacylglycerols accumulation is associated with MASLD through induction of protein kinase C (36).

Further, we found higher levels of isovalerylcarnitine (Car 5:0), a breakdown product of leucine, and L-Palmitoylcarnitine (CAR 16:0), a long chain acyl fatty acid derivative ester of carnitine, in cases compared to controls. Increased acyl carnitines suggest elevated mitochondrial fatty acid oxidation. This is consistent with the concept that MASLD in T1D, like T2D, is associated with both fat synthesis and increased fatty acid oxidation (37), although the later may be only compensatory. Our findings regarding Car 5.0 are consistent with Enooku et al. (38) who measured serum acylcarnitine species in 241 biopsy proven cases of MASLD and reported that acylcarnitine 5.0 positively correlated with steatosis (p = 0.056).

As shown in **Figure 3**, several compounds elevated in our case cohort and (27) were among the top metabolites correlated with steatosis, BMI, and insulin dosing. These metabolites include glutamate, valine, proline, and alanine. The aromatic amino acid, tyrosine was in the top correlating compounds for BMI and insulin dosage while phenylalanine was predictive of steatosis (**Table 4**). Also, we note that the glucose-alanine cycle and alanine metabolism, discussed above in relation to glucose production, were in the top three pathways enriched in cases (**Figure 5**). Selenoamino acid metabolism, the top enriched pathway (**Figure 5**) may be important in preventing hepatic oxidative damage, inflammation, and fibrosis by increasing the activity of glutathione peroxidase (39).

### Limitations

There are several limitations to our current study. Our sample size was small and might have lacked power to detect significant differences between certain metabolites. We acknowledge that a larger study is needed to confirm metabolite perturbations in T1D and for direct comparison to T2D and non-diabetic individuals. But the strength of this study is that we examined a large number of metabolites and inferred pathways in a population (type 1 DM) where little was previously known and provide a basis for future work. Moreover, our metabolite data taken together with our metabolic pathway analyses imply differences between cases and controls despite the multiple listed comparisons. Another limitation is that did not carry out a full lipidomic analysis examining phospholipid subclasses, bile acids, and fatty acid levels, so it is possible that we may have missed other complex lipids that play a significant role in the pathogenesis of MASLD.

We acknowledge that insulin dosing is a surrogate rather than a direct measure of insulin sensitivity. We did not carry out more definitive euglycemic insulin clamp studies due to cost and burden on participants. We emphasize that persons with T1D produce essentially no endogenous insulin. So, unless there are absorption issues or differences in insulin breakdown, the dose required for control is a reasonable approximation of resistance. Of course, this dose needs to be considered relative to control achieved (e.g., HbA1c). But since cases took more insulin despite higher HbA1c levels it could be argued that they would have required even more insulin than controls if control were equalized.

The FibroScan is limited in detecting MASLD in participants with BMI >35 kg/m2 (40) so there could have been some overestimation of steatosis and fibrosis in some participants. We minimized this by using the XL probes for obese patients, a technique found to be more accurate for detecting greater than stage F2 fibrosis and cirrhosis (41). Our case and control groups were studied at about the same age, although the control group had a longer mean duration of diabetes. It is difficult to interpret this as related to steatosis and fibrosis scores, although the data is surprising in that, intuitively, we might expect that longer duration would lead to worse scores. Since our controls had lower scores, we might speculate that the differences between cases and controls would have been greater if durations were equal.

## Conclusion

Our data support the concept that MASLD coincident with T1D is a distinctive health complication. Our data suggest that among individuals with T1D, MASLD is associated with features of insulin resistance and corresponding metabolite perturbations. Future work is needed to better characterize the pathways that contribute to the development and severity of MASLD in T1D and provide rationale for therapeutic interventions that might prevent or treat MASLD in T1D.

## Data Availability

The datasets presented in this study can be found in online repositories. The names of the repository/repositories and accession number(s) can be found in the article/**Supplementary Material**.
