# Supplementary material for: Metabolite perturbations in type 1 diabetes associated with metabolic dysfunction-associated steatotic liver disease
Source: Front Endocrinol (Lausanne). 2025 Jun 3;16:1500242. doi: 10.3389/fendo.2025.1500242 (PMC12188458; doi:10.3389/fendo.2025.1500242)
Supplement: Supplementary file 1 [file Presentation1.pptx]

## Slide 1
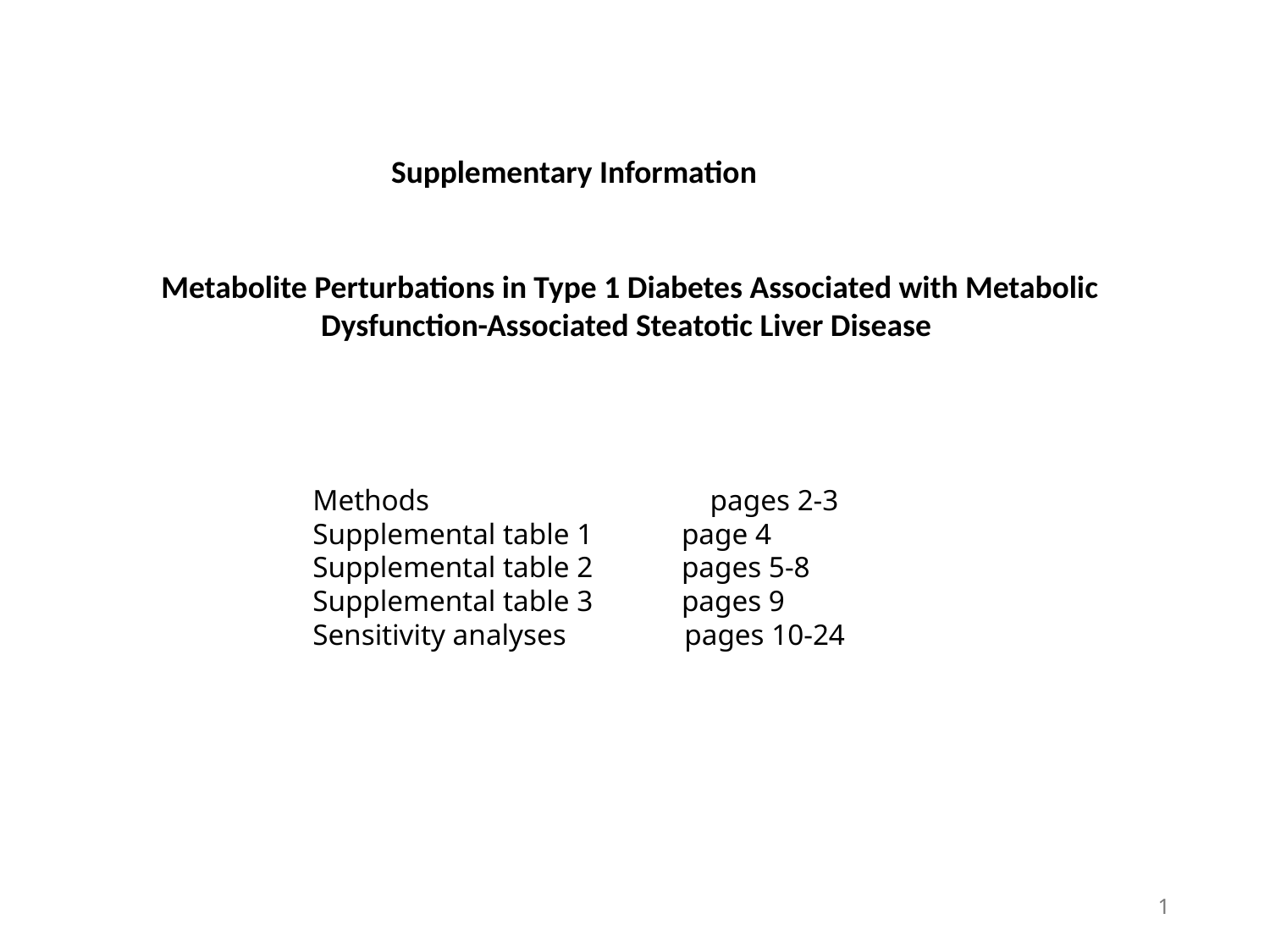

Supplementary Information
Metabolite Perturbations in Type 1 Diabetes Associated with Metabolic Dysfunction-Associated Steatotic Liver Disease
Methods pages 2-3
Supplemental table 1 page 4
Supplemental table 2 pages 5-8
Supplemental table 3 pages 9
Sensitivity analyses pages 10-24
1

## Slide 2
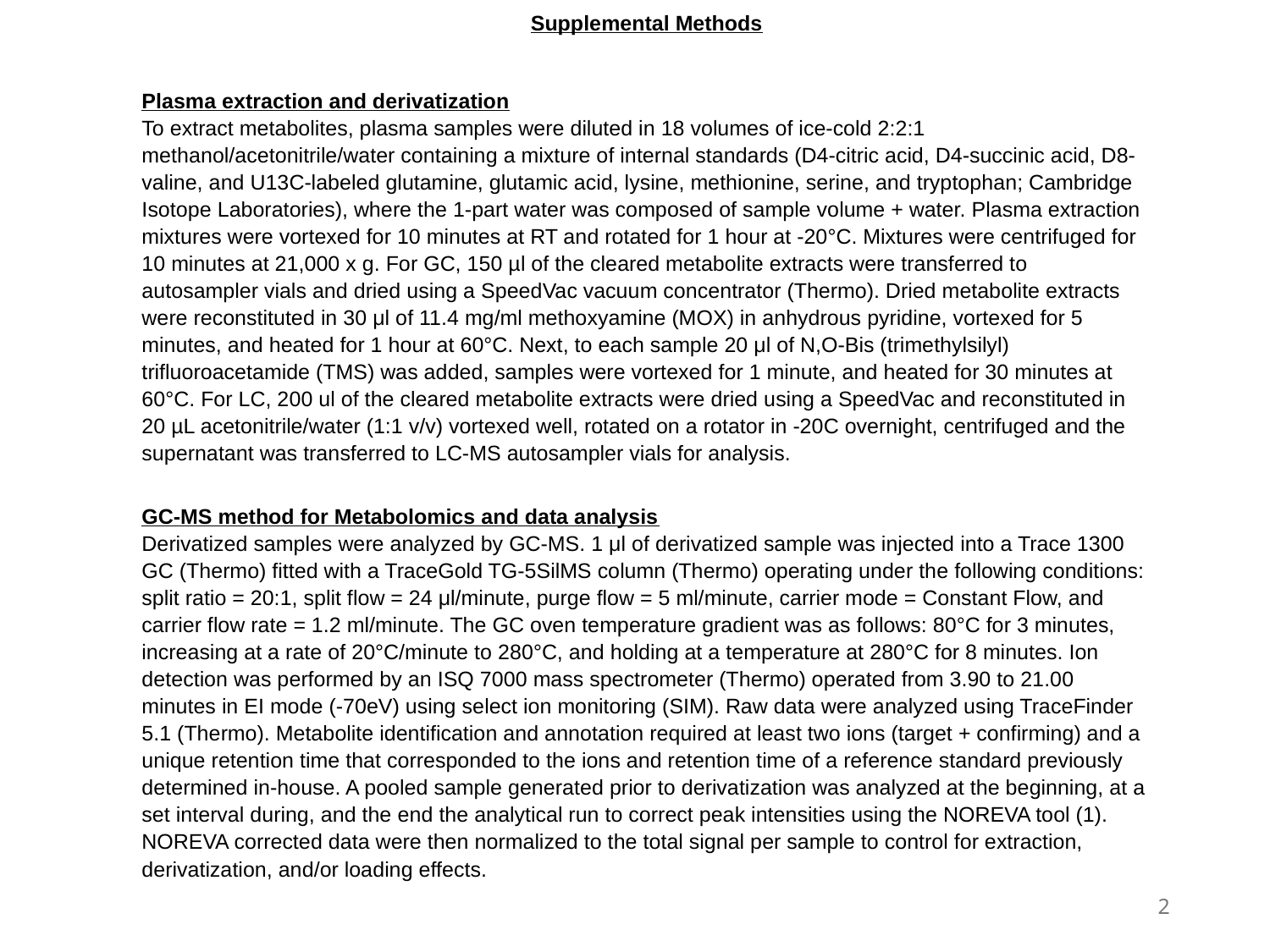

Supplemental Methods
Plasma extraction and derivatization
To extract metabolites, plasma samples were diluted in 18 volumes of ice-cold 2:2:1 methanol/acetonitrile/water containing a mixture of internal standards (D4-citric acid, D4-succinic acid, D8-valine, and U13C-labeled glutamine, glutamic acid, lysine, methionine, serine, and tryptophan; Cambridge Isotope Laboratories), where the 1-part water was composed of sample volume + water. Plasma extraction mixtures were vortexed for 10 minutes at RT and rotated for 1 hour at -20°C. Mixtures were centrifuged for 10 minutes at 21,000 x g. For GC, 150 µl of the cleared metabolite extracts were transferred to autosampler vials and dried using a SpeedVac vacuum concentrator (Thermo). Dried metabolite extracts were reconstituted in 30 μl of 11.4 mg/ml methoxyamine (MOX) in anhydrous pyridine, vortexed for 5 minutes, and heated for 1 hour at 60°C. Next, to each sample 20 μl of N,O-Bis (trimethylsilyl) trifluoroacetamide (TMS) was added, samples were vortexed for 1 minute, and heated for 30 minutes at 60°C. For LC, 200 ul of the cleared metabolite extracts were dried using a SpeedVac and reconstituted in 20 µL acetonitrile/water (1:1 v/v) vortexed well, rotated on a rotator in -20C overnight, centrifuged and the supernatant was transferred to LC-MS autosampler vials for analysis.
GC-MS method for Metabolomics and data analysis
Derivatized samples were analyzed by GC-MS. 1 μl of derivatized sample was injected into a Trace 1300 GC (Thermo) fitted with a TraceGold TG-5SilMS column (Thermo) operating under the following conditions: split ratio = 20:1, split flow = 24 μl/minute, purge flow = 5 ml/minute, carrier mode = Constant Flow, and carrier flow rate = 1.2 ml/minute. The GC oven temperature gradient was as follows: 80°C for 3 minutes, increasing at a rate of 20°C/minute to 280°C, and holding at a temperature at 280°C for 8 minutes. Ion detection was performed by an ISQ 7000 mass spectrometer (Thermo) operated from 3.90 to 21.00 minutes in EI mode (-70eV) using select ion monitoring (SIM). Raw data were analyzed using TraceFinder 5.1 (Thermo). Metabolite identification and annotation required at least two ions (target + confirming) and a unique retention time that corresponded to the ions and retention time of a reference standard previously determined in-house. A pooled sample generated prior to derivatization was analyzed at the beginning, at a set interval during, and the end the analytical run to correct peak intensities using the NOREVA tool (1). NOREVA corrected data were then normalized to the total signal per sample to control for extraction, derivatization, and/or loading effects.
2

## Slide 3
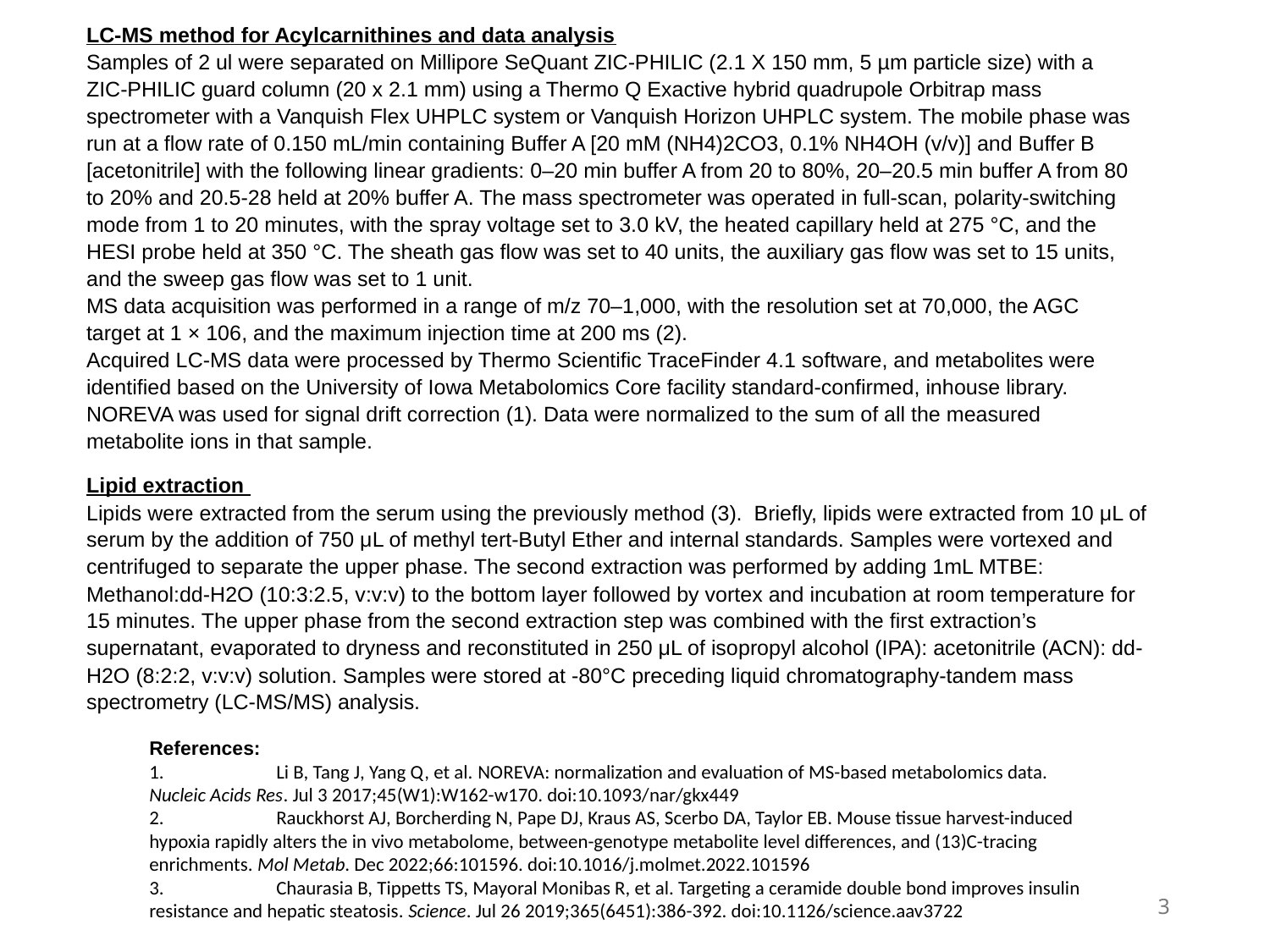

LC-MS method for Acylcarnithines and data analysis
Samples of 2 ul were separated on Millipore SeQuant ZIC-PHILIC (2.1 X 150 mm, 5 µm particle size) with a ZIC-PHILIC guard column (20 x 2.1 mm) using a Thermo Q Exactive hybrid quadrupole Orbitrap mass spectrometer with a Vanquish Flex UHPLC system or Vanquish Horizon UHPLC system. The mobile phase was run at a flow rate of 0.150 mL/min containing Buffer A [20 mM (NH4)2CO3, 0.1% NH4OH (v/v)] and Buffer B [acetonitrile] with the following linear gradients: 0–20 min buffer A from 20 to 80%, 20–20.5 min buffer A from 80 to 20% and 20.5-28 held at 20% buffer A. The mass spectrometer was operated in full-scan, polarity-switching mode from 1 to 20 minutes, with the spray voltage set to 3.0 kV, the heated capillary held at 275 °C, and the HESI probe held at 350 °C. The sheath gas flow was set to 40 units, the auxiliary gas flow was set to 15 units, and the sweep gas flow was set to 1 unit.
MS data acquisition was performed in a range of m/z 70–1,000, with the resolution set at 70,000, the AGC target at 1 × 10^6^, and the maximum injection time at 200 ms (2).
Acquired LC-MS data were processed by Thermo Scientific TraceFinder 4.1 software, and metabolites were identified based on the University of Iowa Metabolomics Core facility standard-confirmed, inhouse library. NOREVA was used for signal drift correction (1). Data were normalized to the sum of all the measured metabolite ions in that sample.
Lipid extraction
Lipids were extracted from the serum using the previously method (3). Briefly, lipids were extracted from 10 μL of serum by the addition of 750 μL of methyl tert-Butyl Ether and internal standards. Samples were vortexed and centrifuged to separate the upper phase. The second extraction was performed by adding 1mL MTBE: Methanol:dd-H2O (10:3:2.5, v:v:v) to the bottom layer followed by vortex and incubation at room temperature for 15 minutes. The upper phase from the second extraction step was combined with the first extraction’s supernatant, evaporated to dryness and reconstituted in 250 μL of isopropyl alcohol (IPA): acetonitrile (ACN): dd-H2O (8:2:2, v:v:v) solution. Samples were stored at -80°C preceding liquid chromatography-tandem mass spectrometry (LC-MS/MS) analysis.
References:
1.	Li B, Tang J, Yang Q, et al. NOREVA: normalization and evaluation of MS-based metabolomics data. Nucleic Acids Res. Jul 3 2017;45(W1):W162-w170. doi:10.1093/nar/gkx449
2.	Rauckhorst AJ, Borcherding N, Pape DJ, Kraus AS, Scerbo DA, Taylor EB. Mouse tissue harvest-induced hypoxia rapidly alters the in vivo metabolome, between-genotype metabolite level differences, and (13)C-tracing enrichments. Mol Metab. Dec 2022;66:101596. doi:10.1016/j.molmet.2022.101596
3.	Chaurasia B, Tippetts TS, Mayoral Monibas R, et al. Targeting a ceramide double bond improves insulin resistance and hepatic steatosis. Science. Jul 26 2019;365(6451):386-392. doi:10.1126/science.aav3722
3

## Slide 4
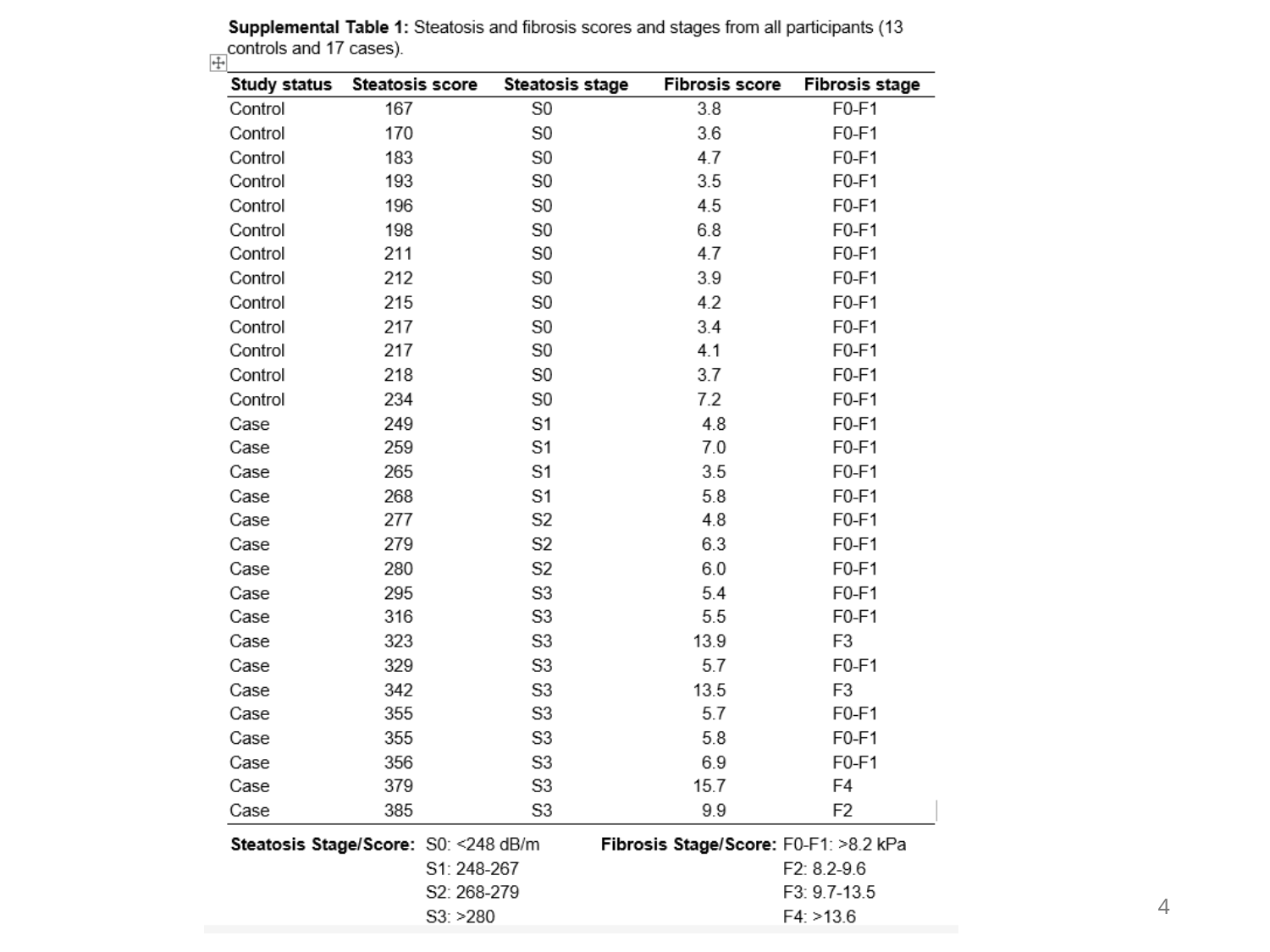

4

## Slide 5
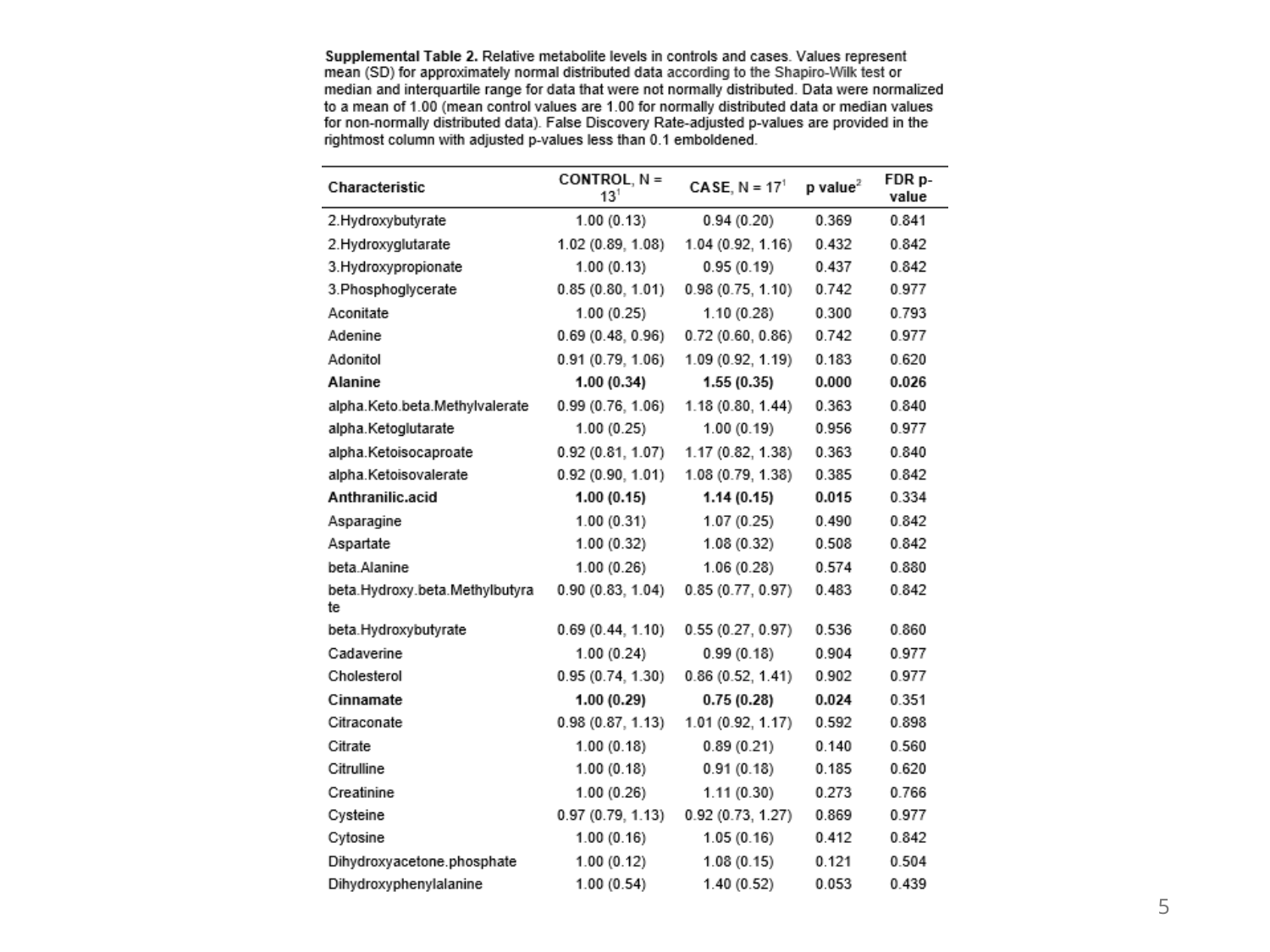

5

## Slide 6
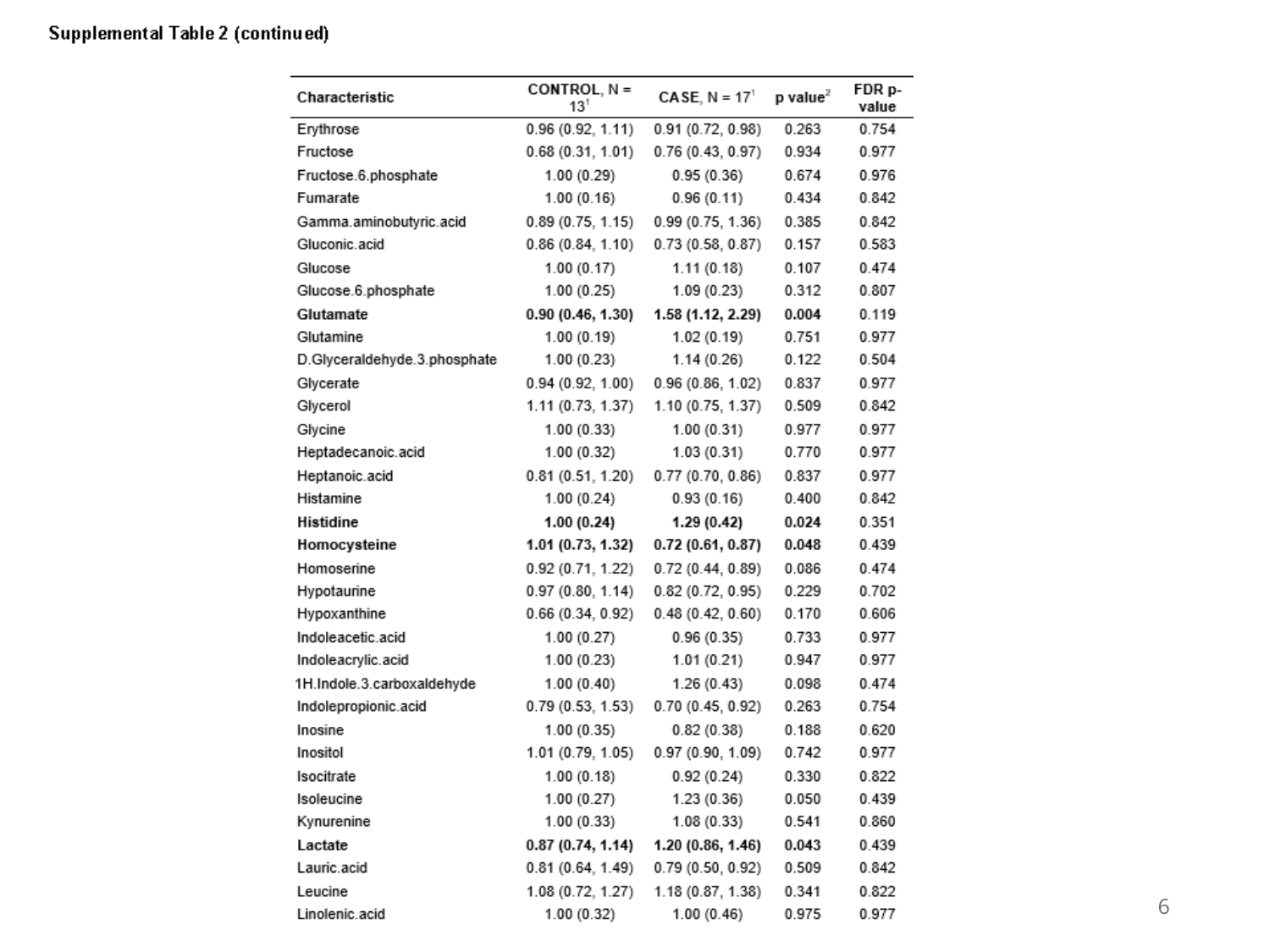

6

## Slide 7
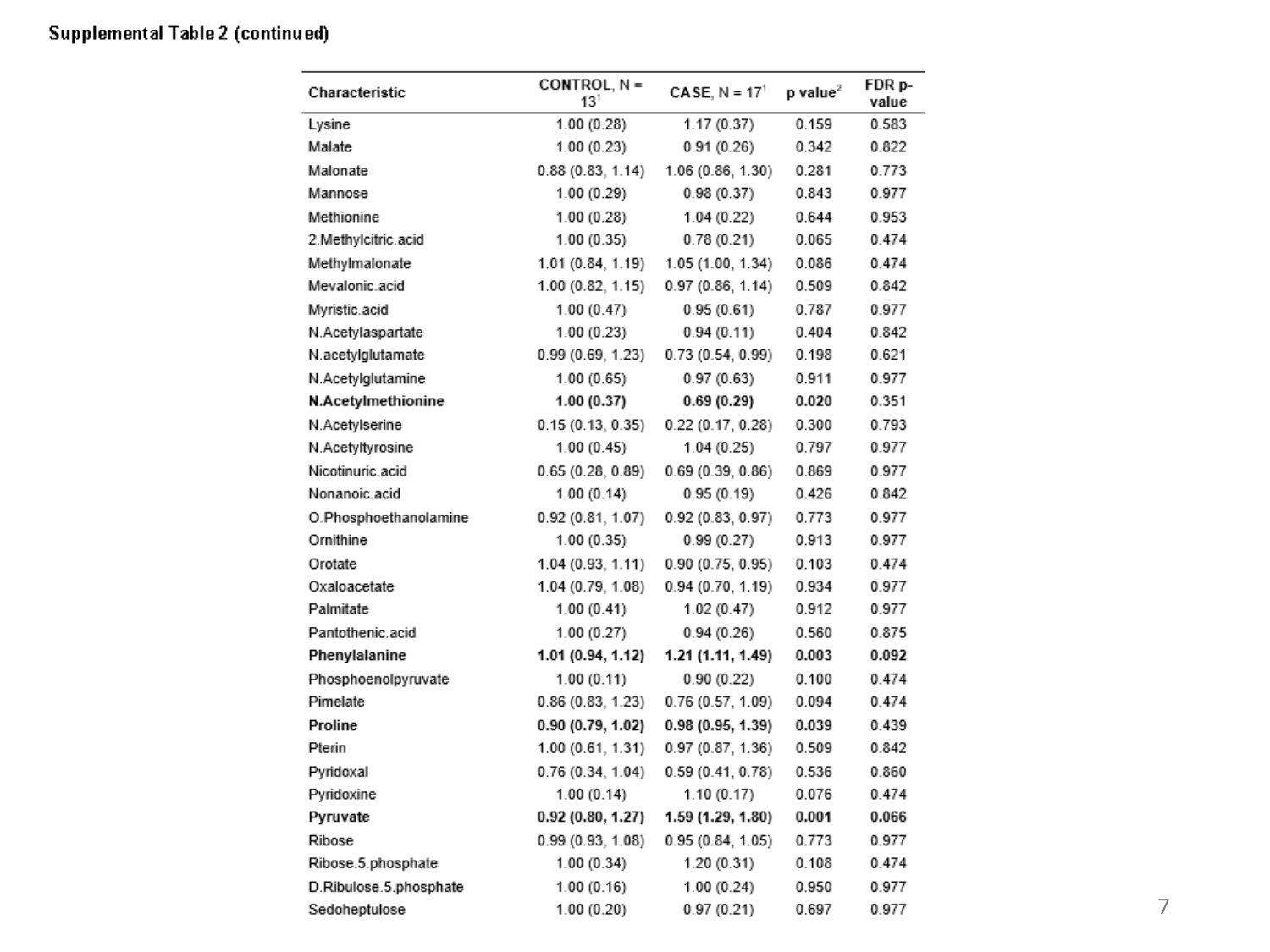

7

## Slide 8
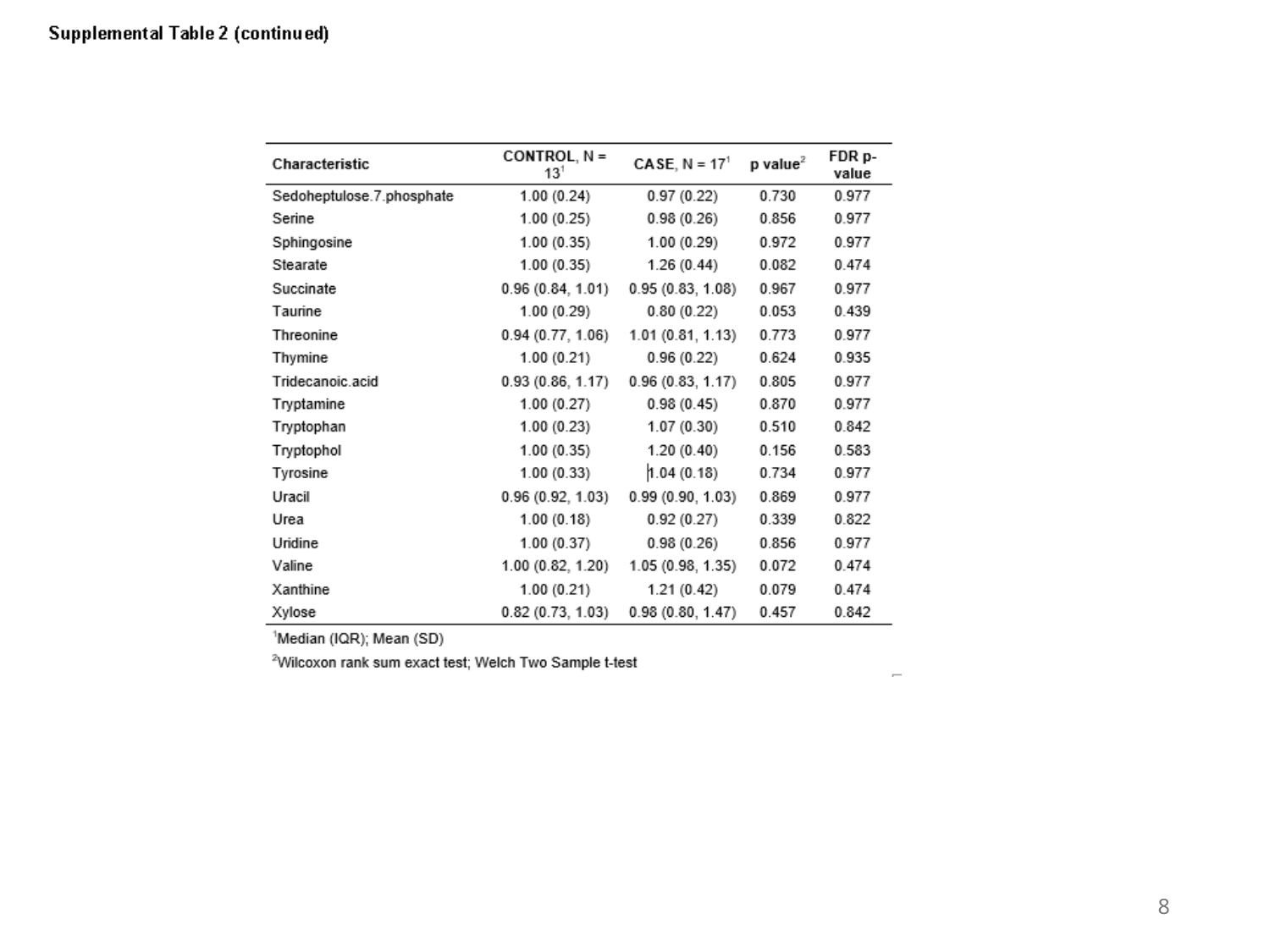

8

## Slide 9
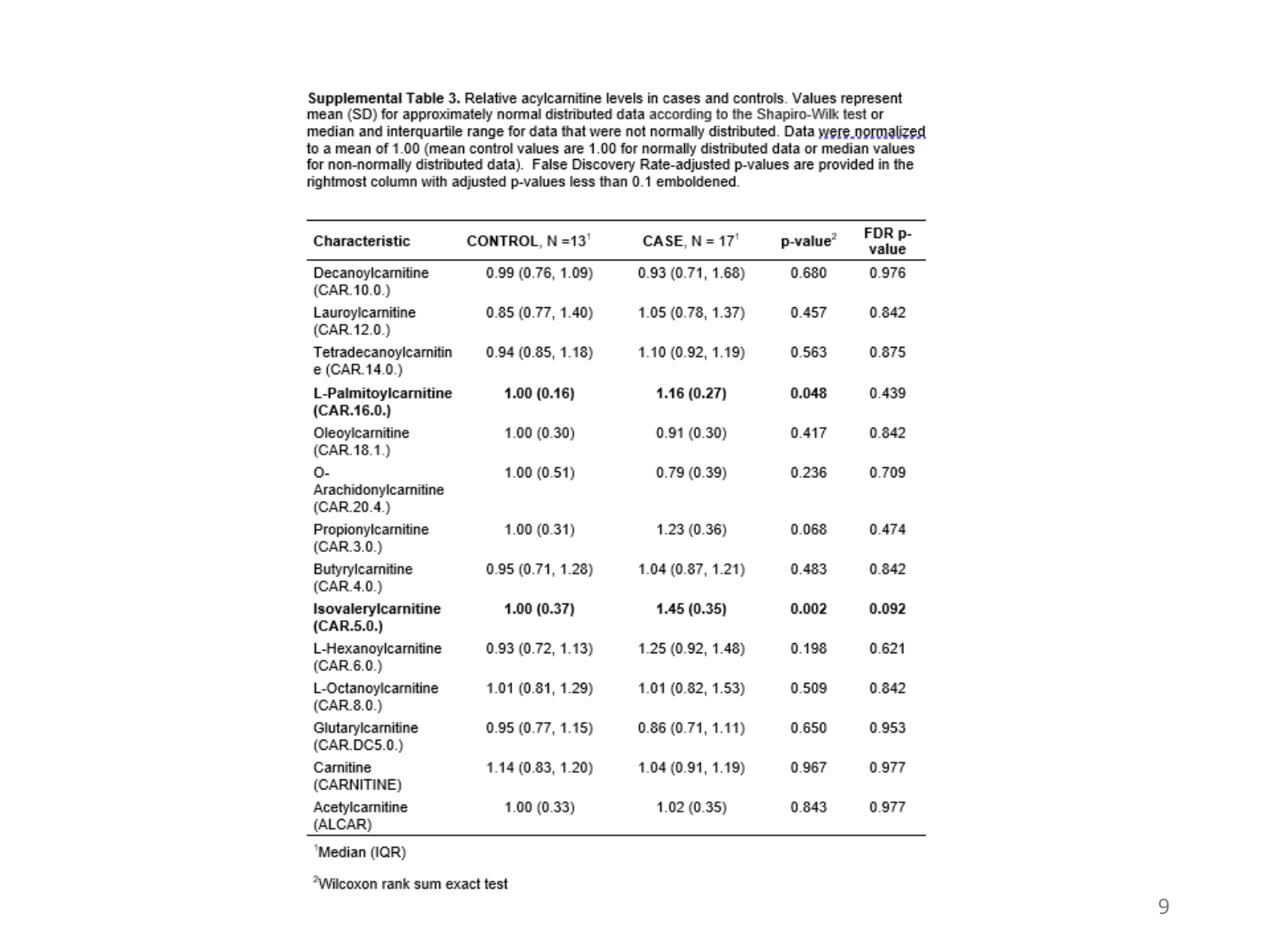

9

## Slide 10
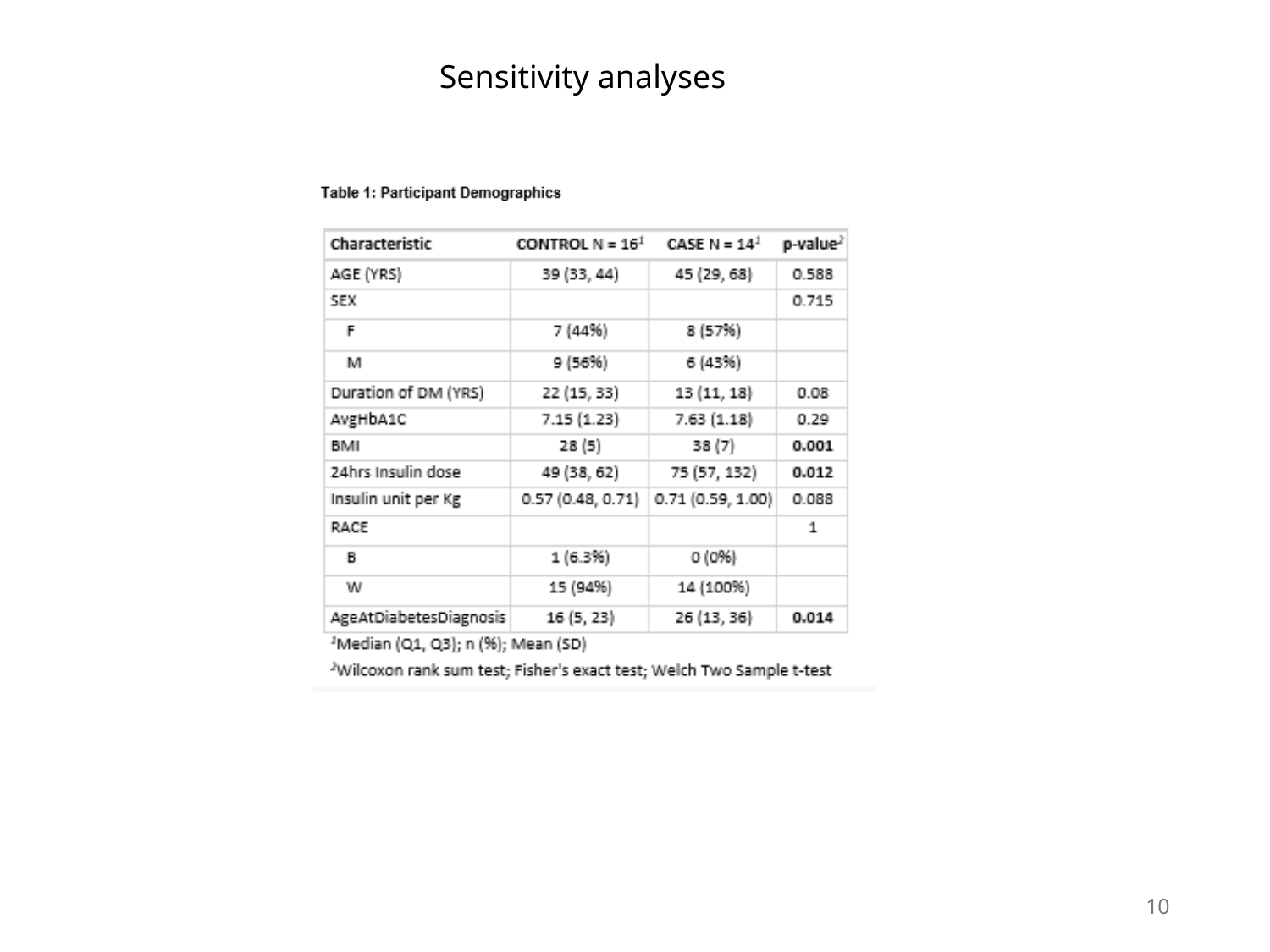

Sensitivity analyses
10

## Slide 11
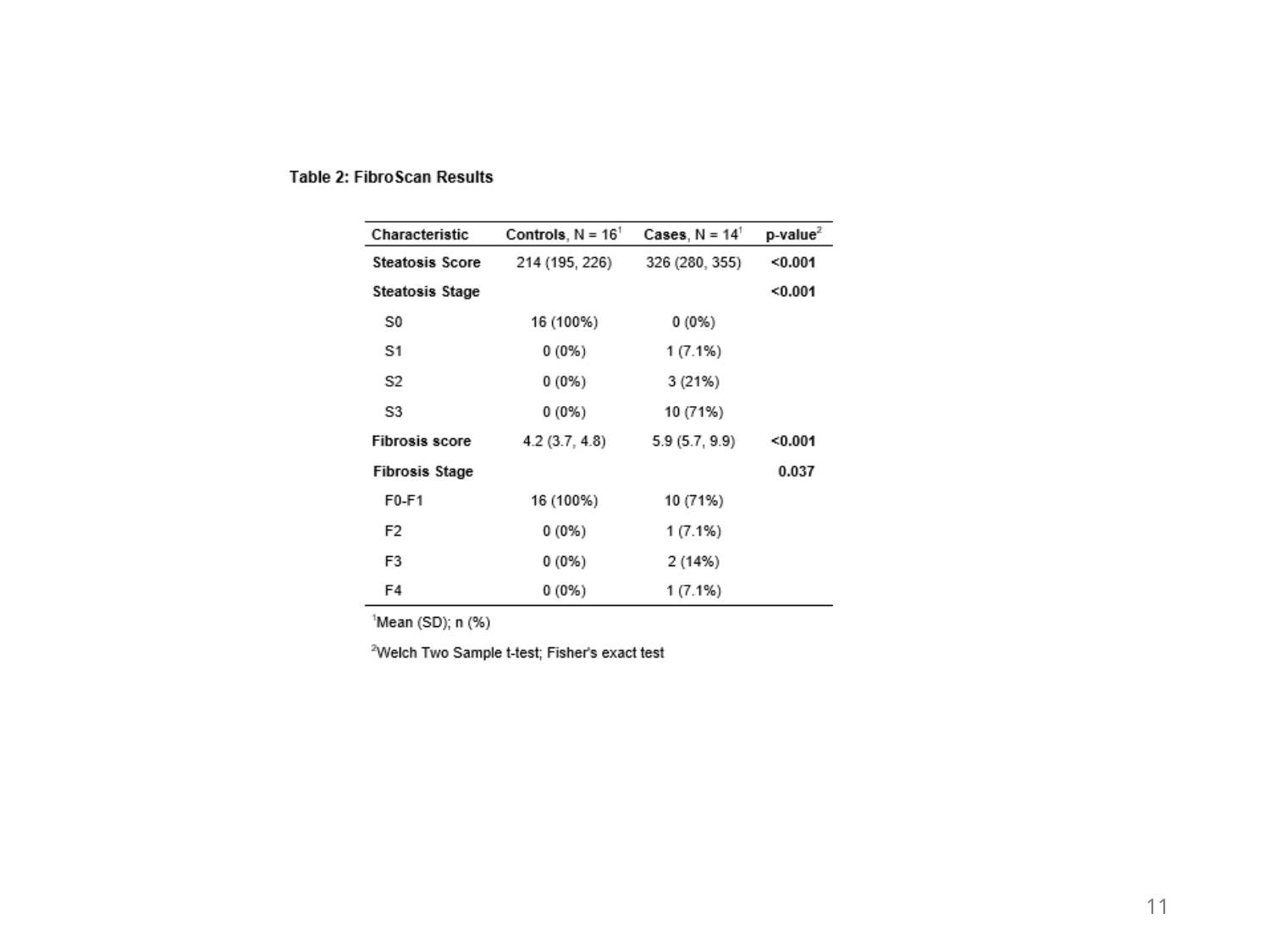

11

## Slide 12
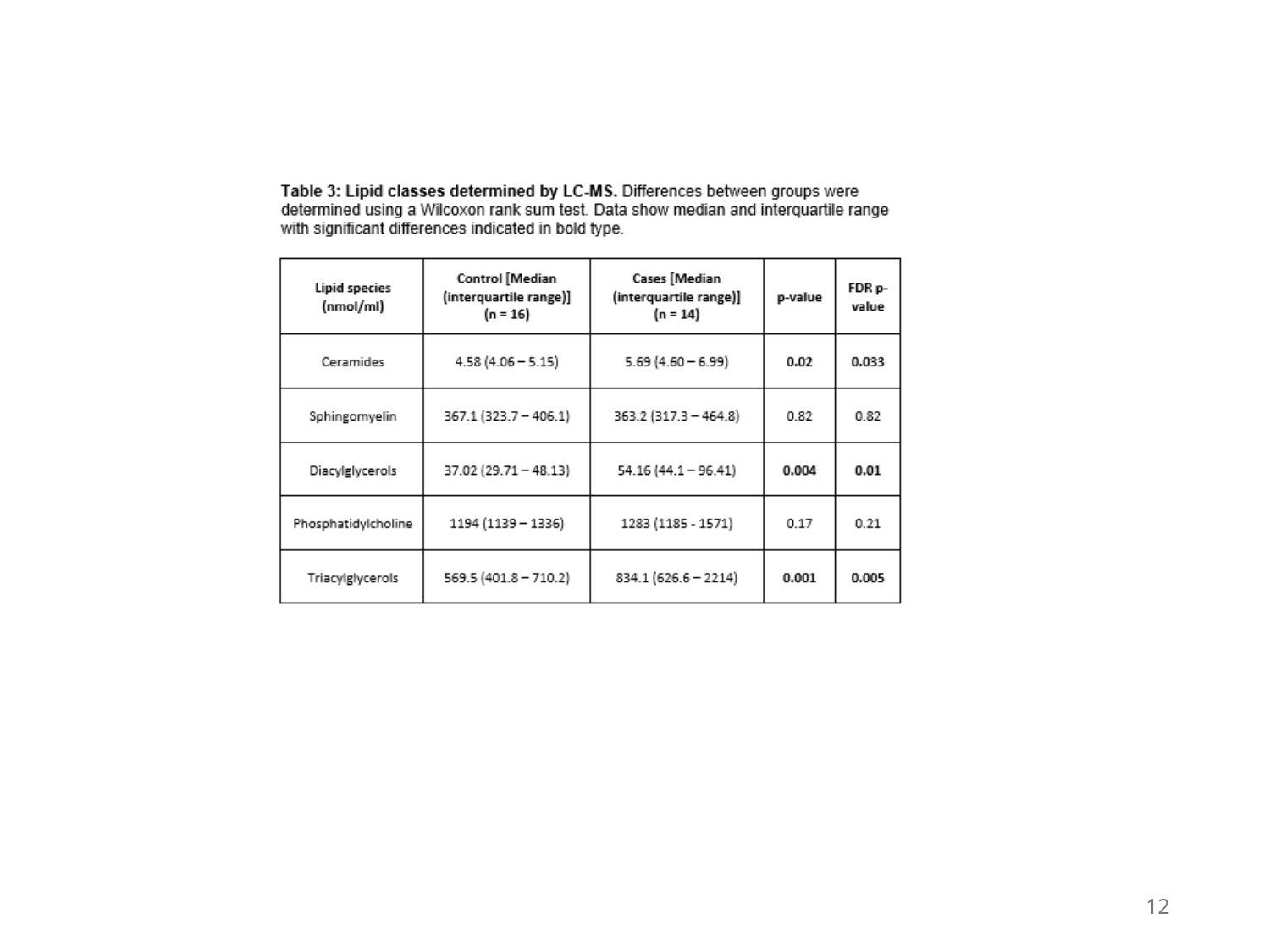

12

## Slide 13
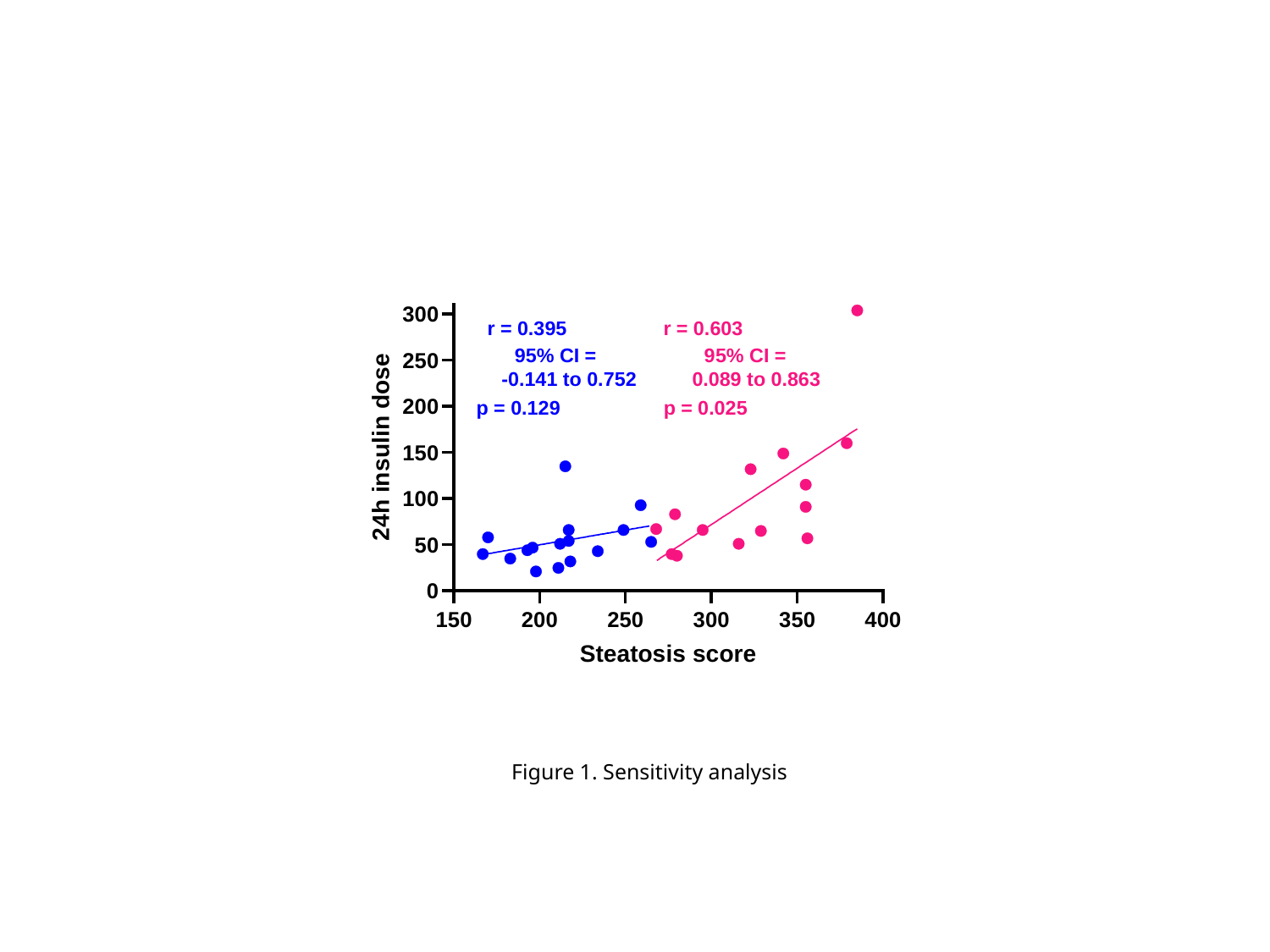

Figure 1. Sensitivity analysis

## Slide 14
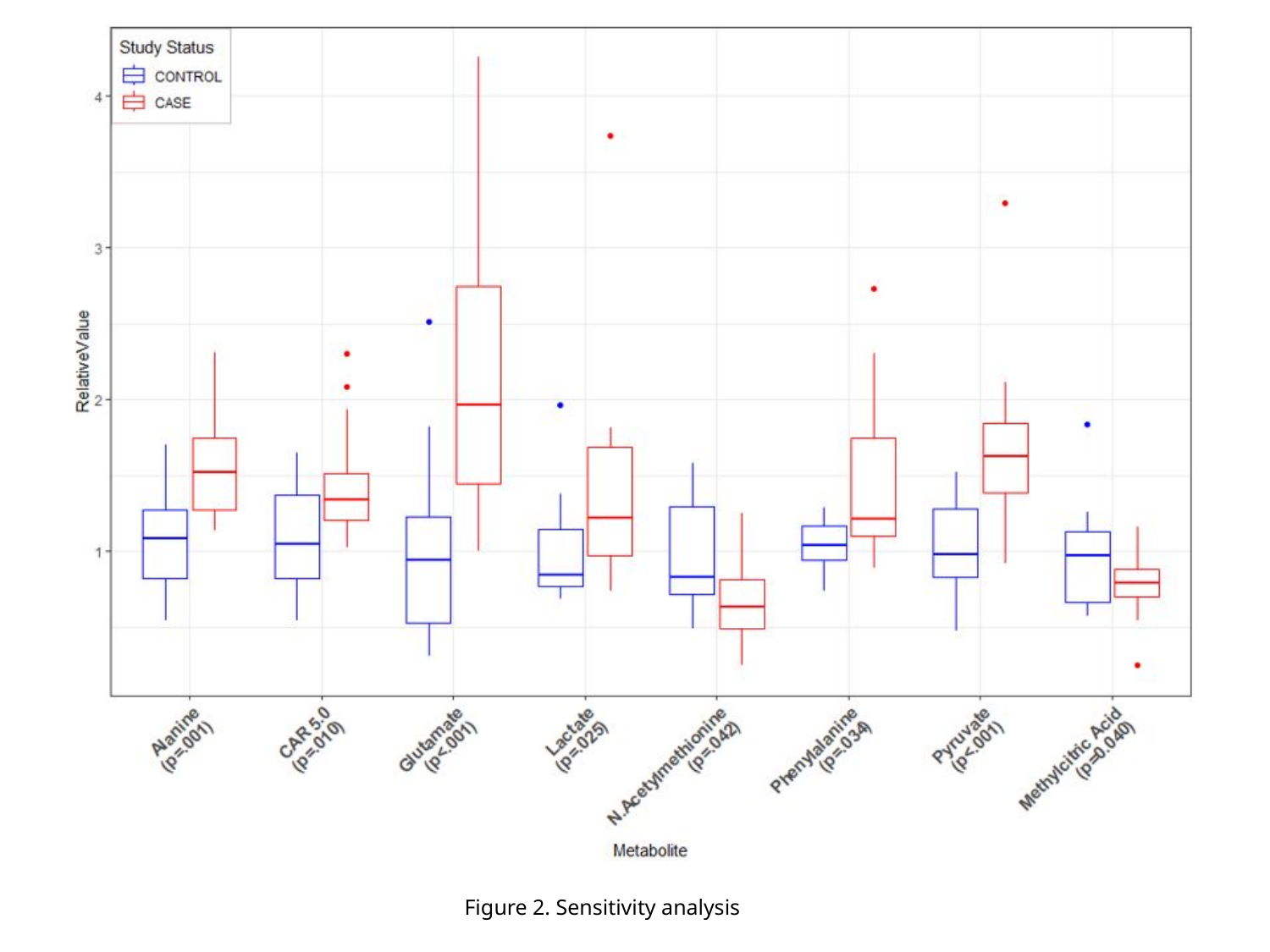

Figure 2. Sensitivity analysis

## Slide 15
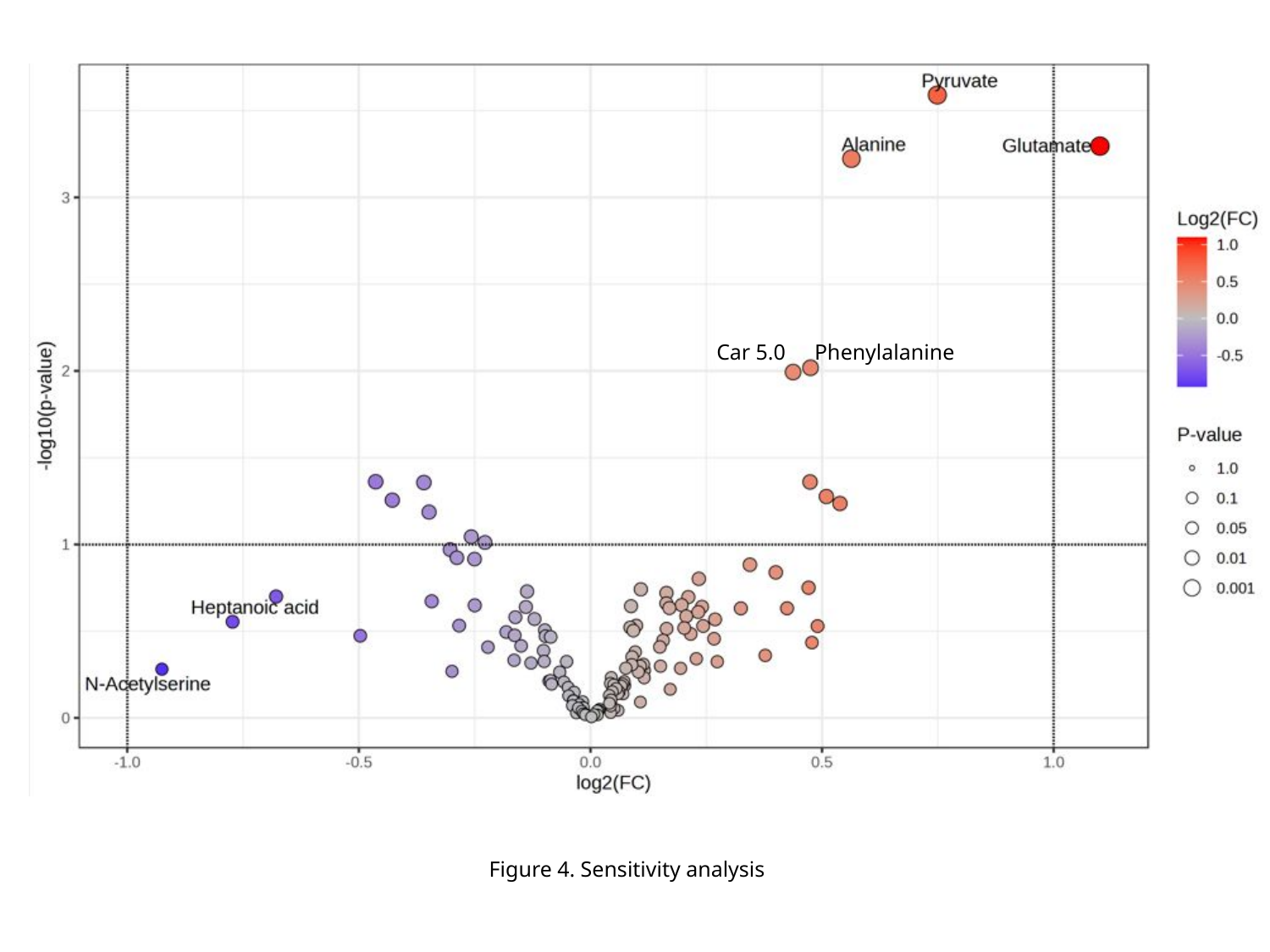

Car 5.0
Phenylalanine
Figure 4. Sensitivity analysis

## Slide 16
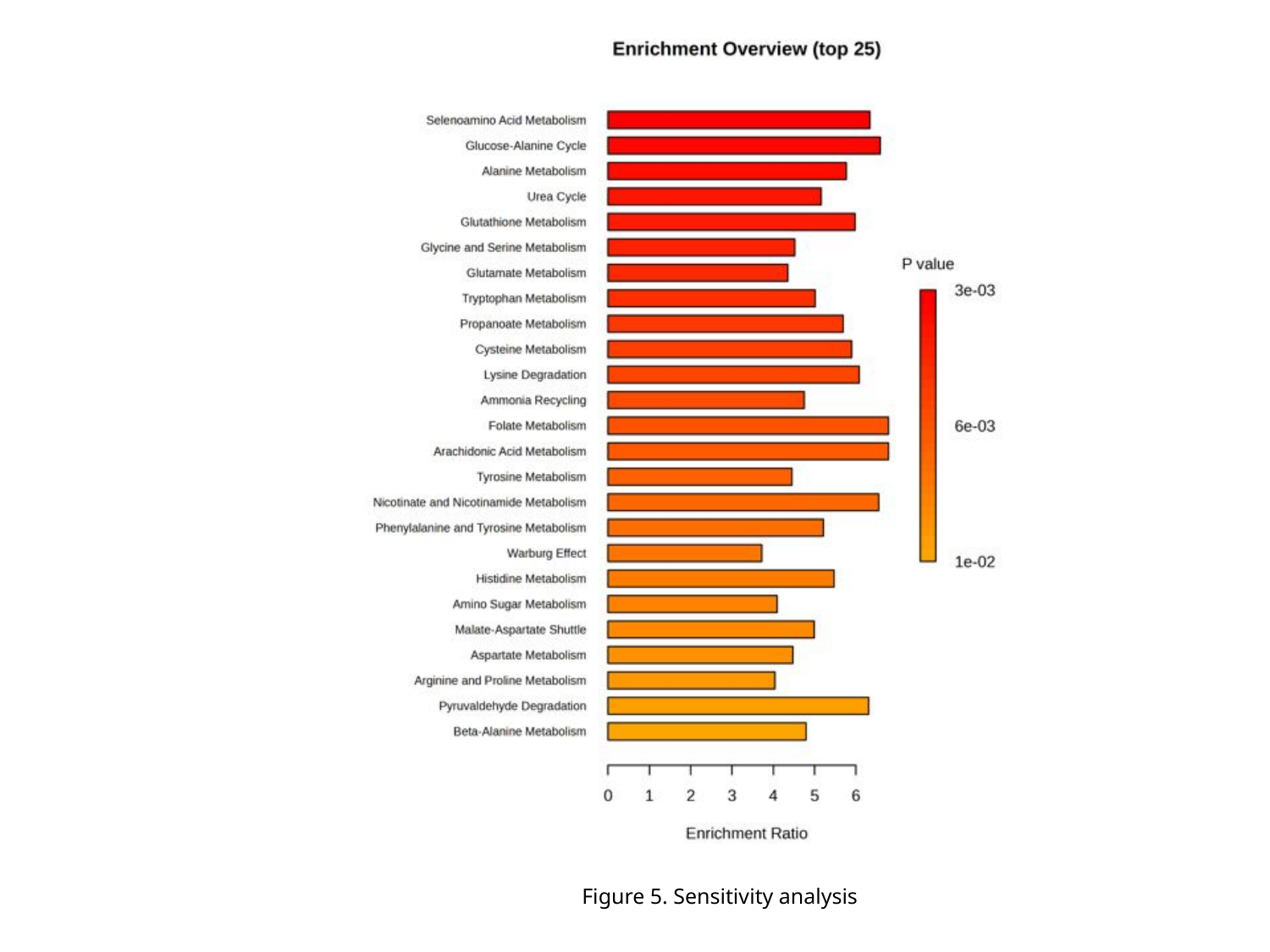

Figure 5. Sensitivity analysis

## Slide 17
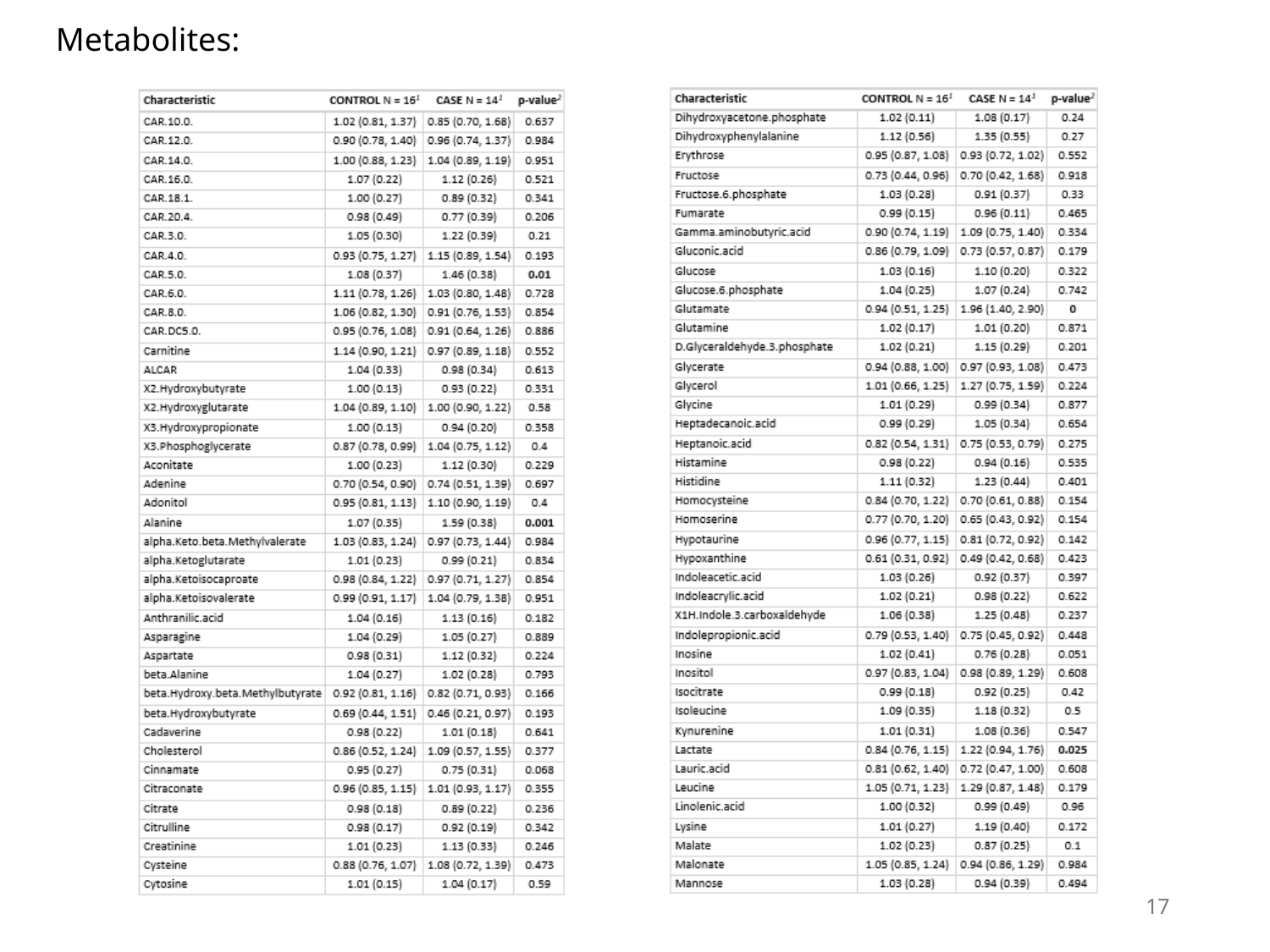

Metabolites:
17

## Slide 18
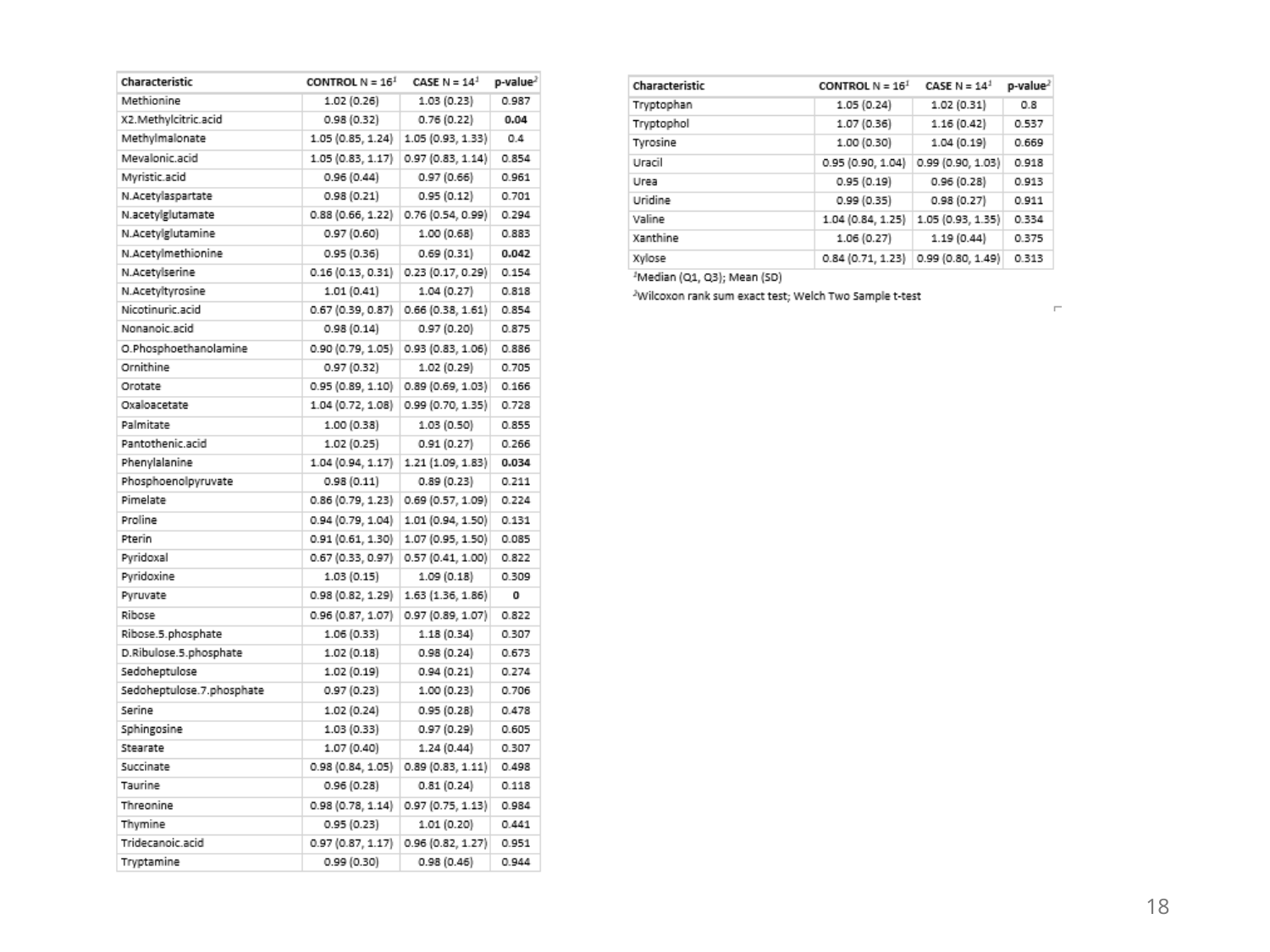

18

## Slide 19
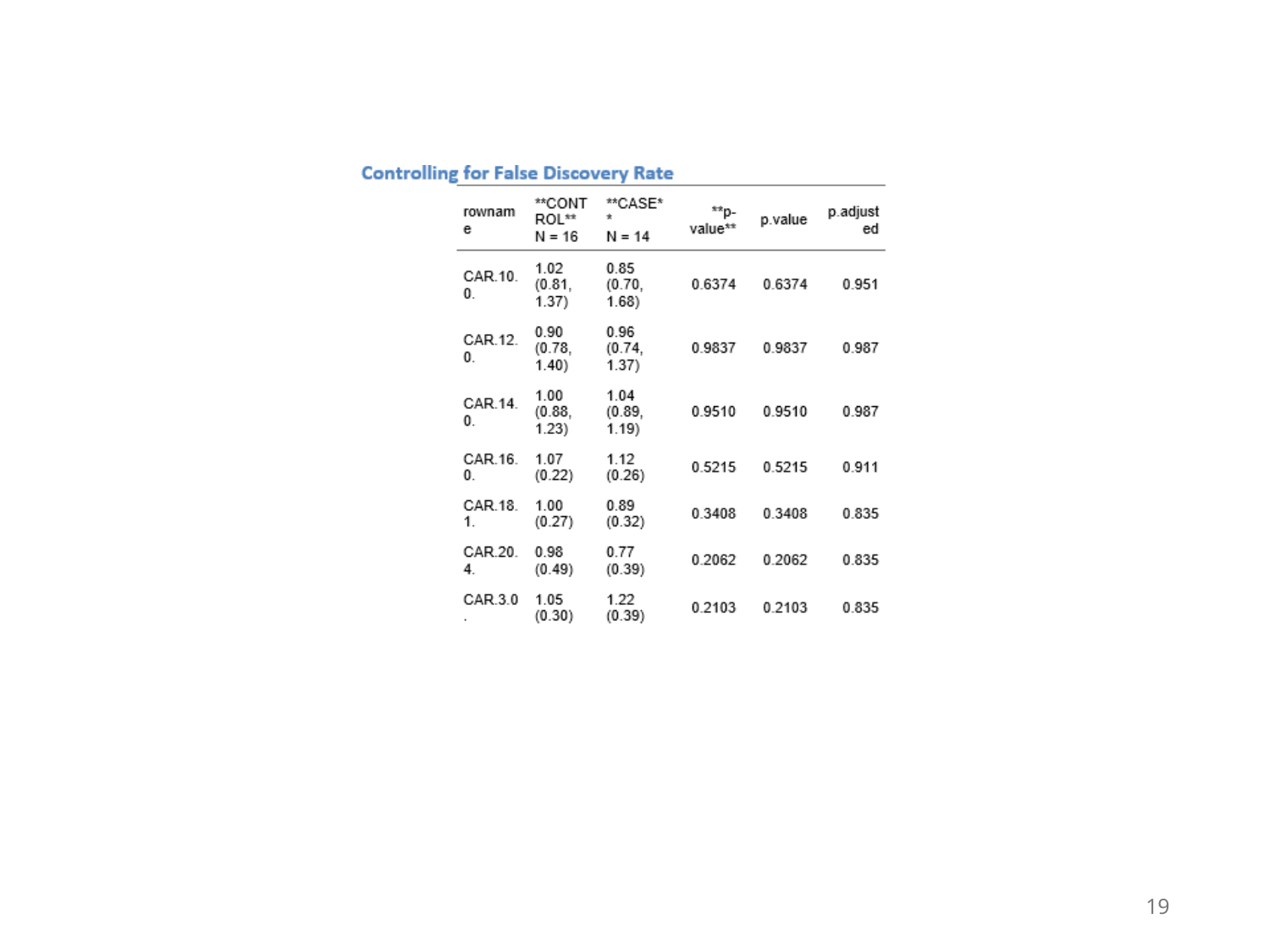

19

## Slide 20
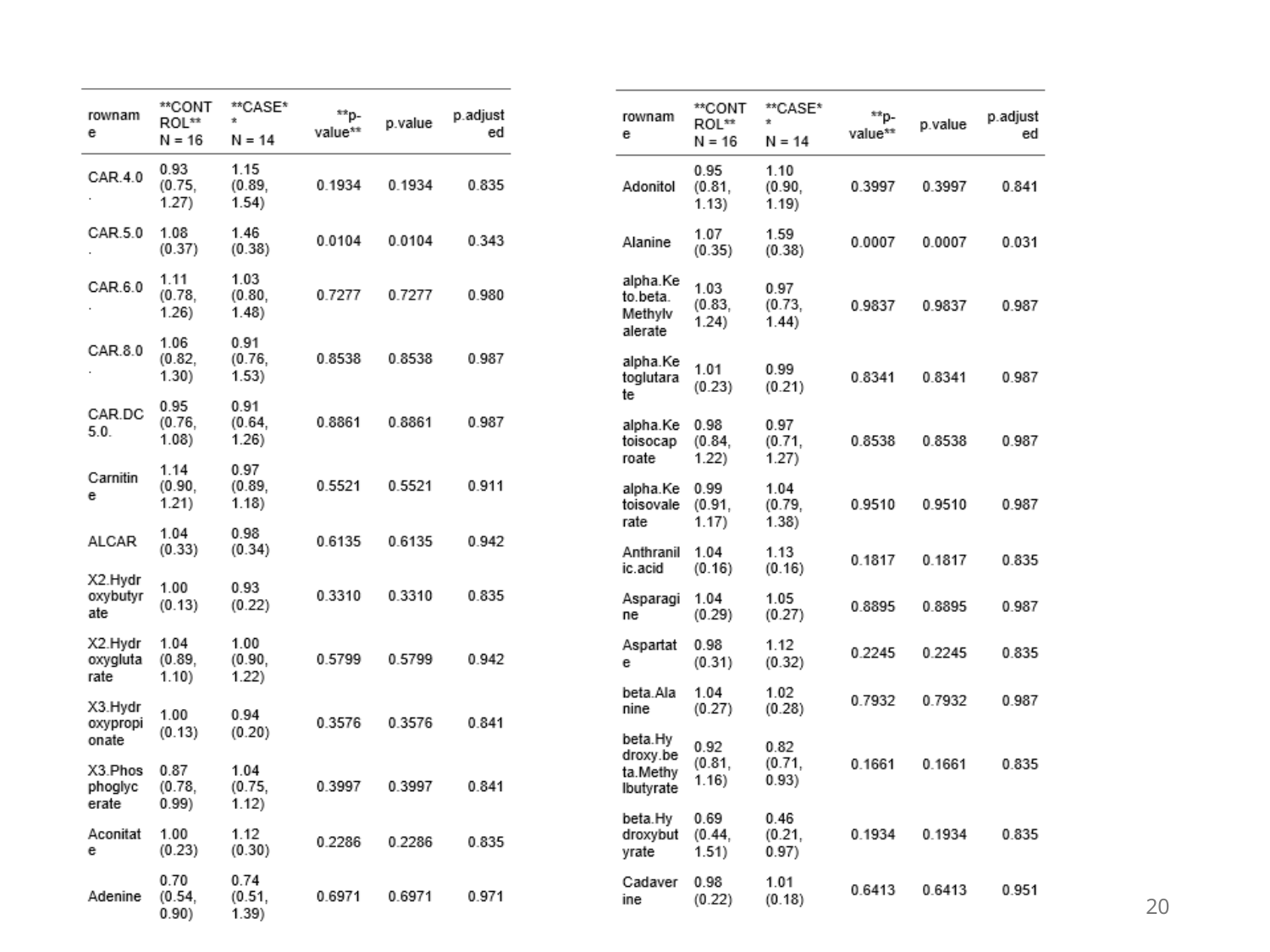

20

## Slide 21
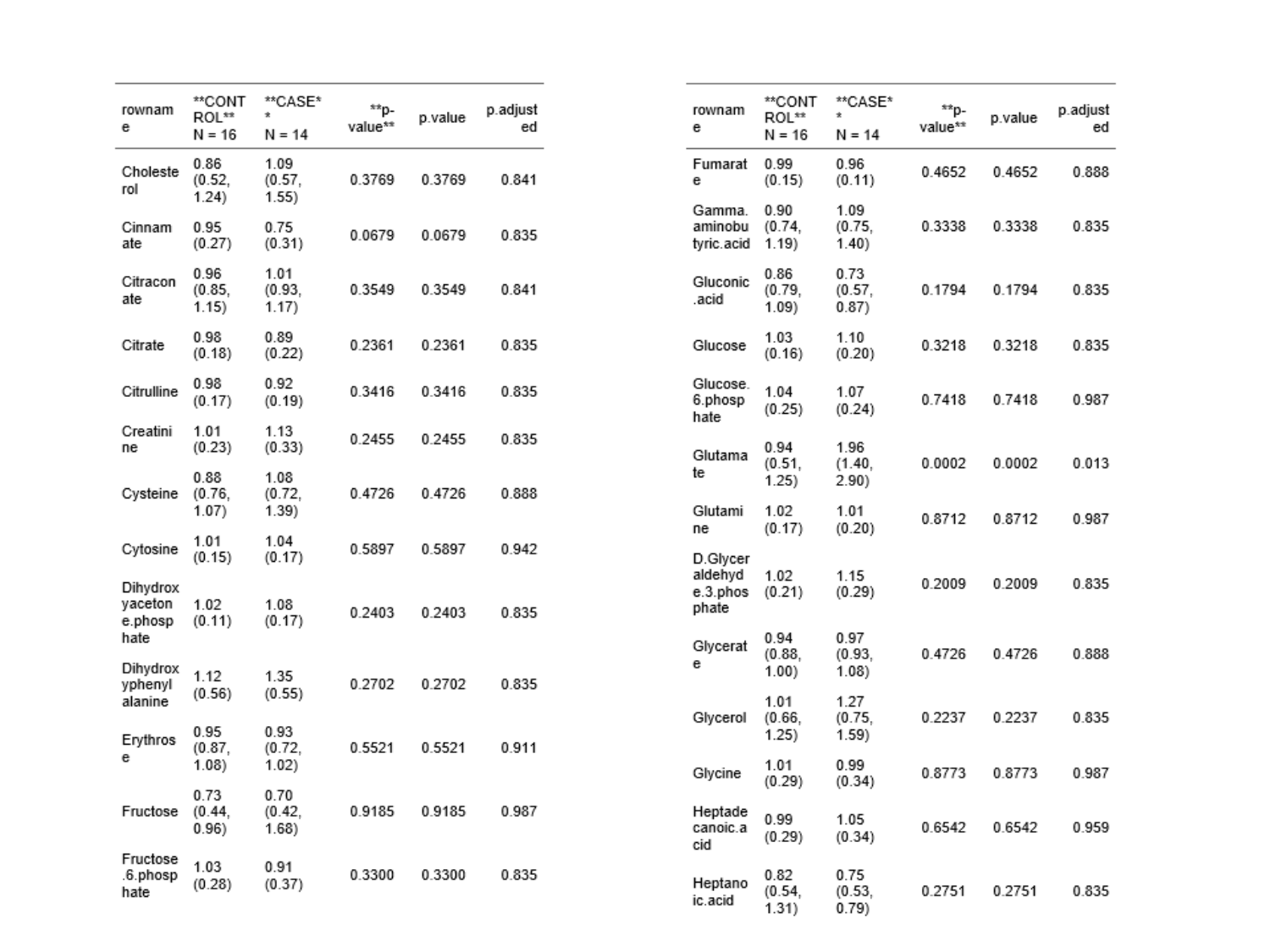

21

## Slide 22
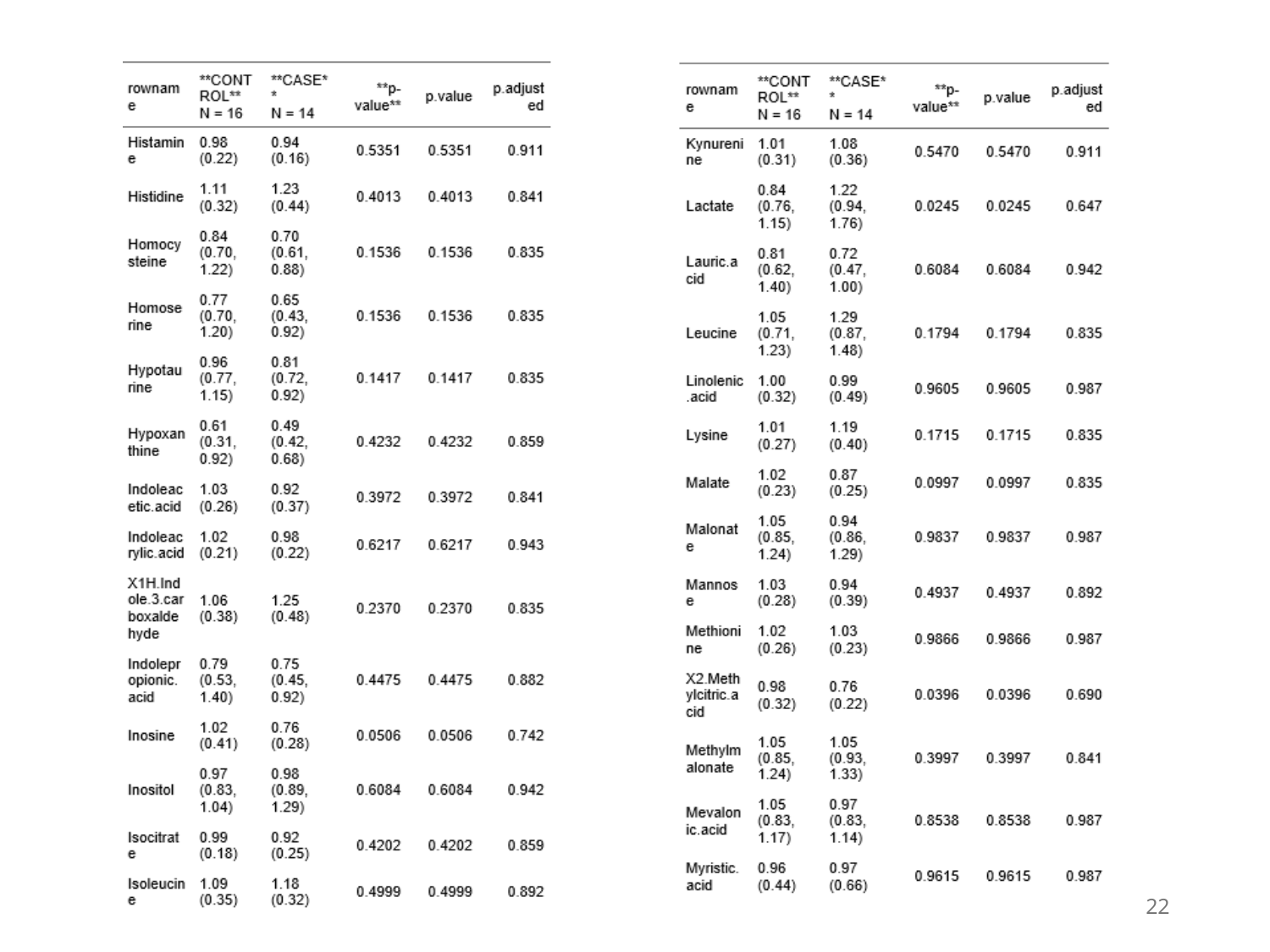

22

## Slide 23
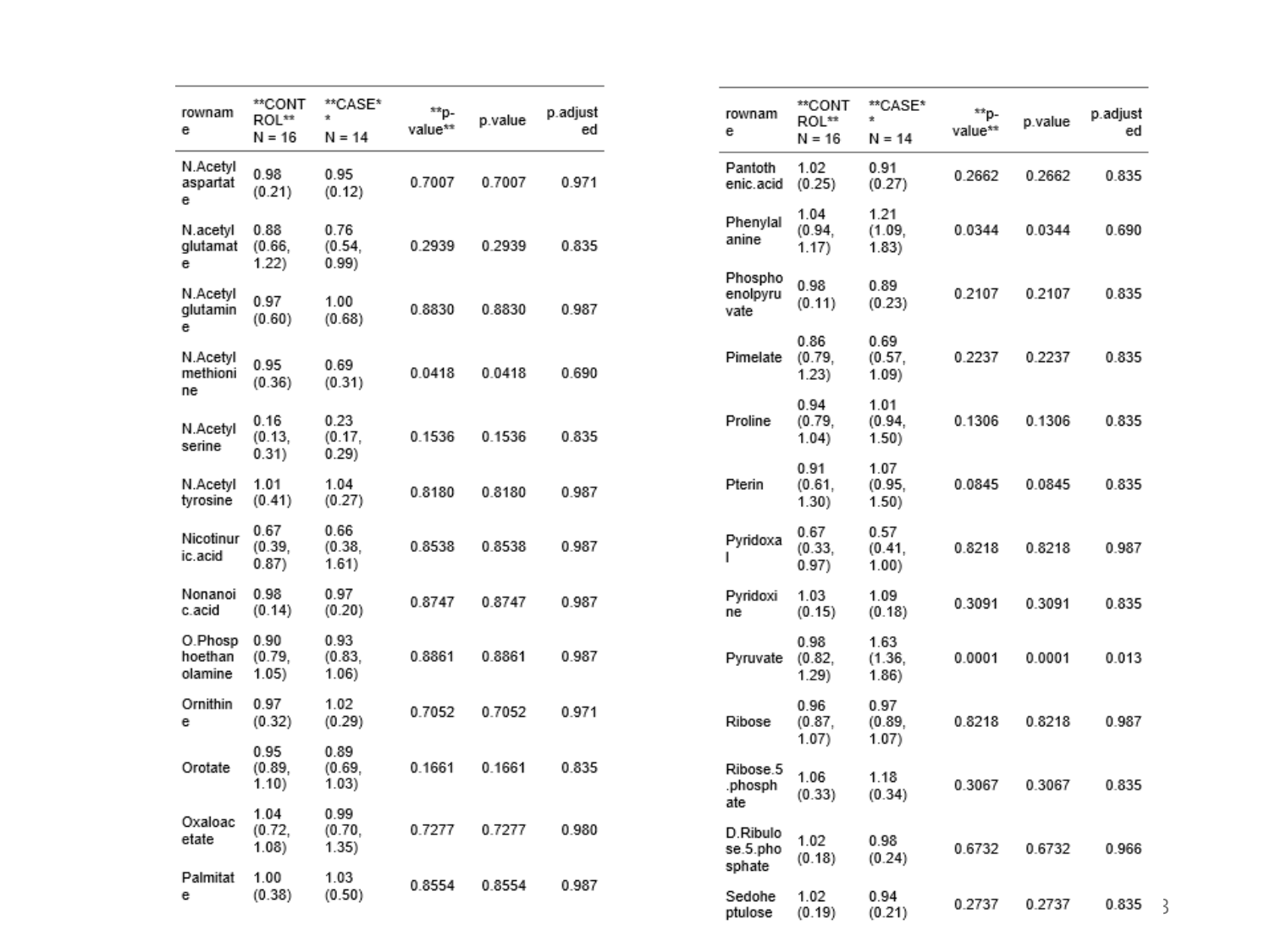

23

## Slide 24
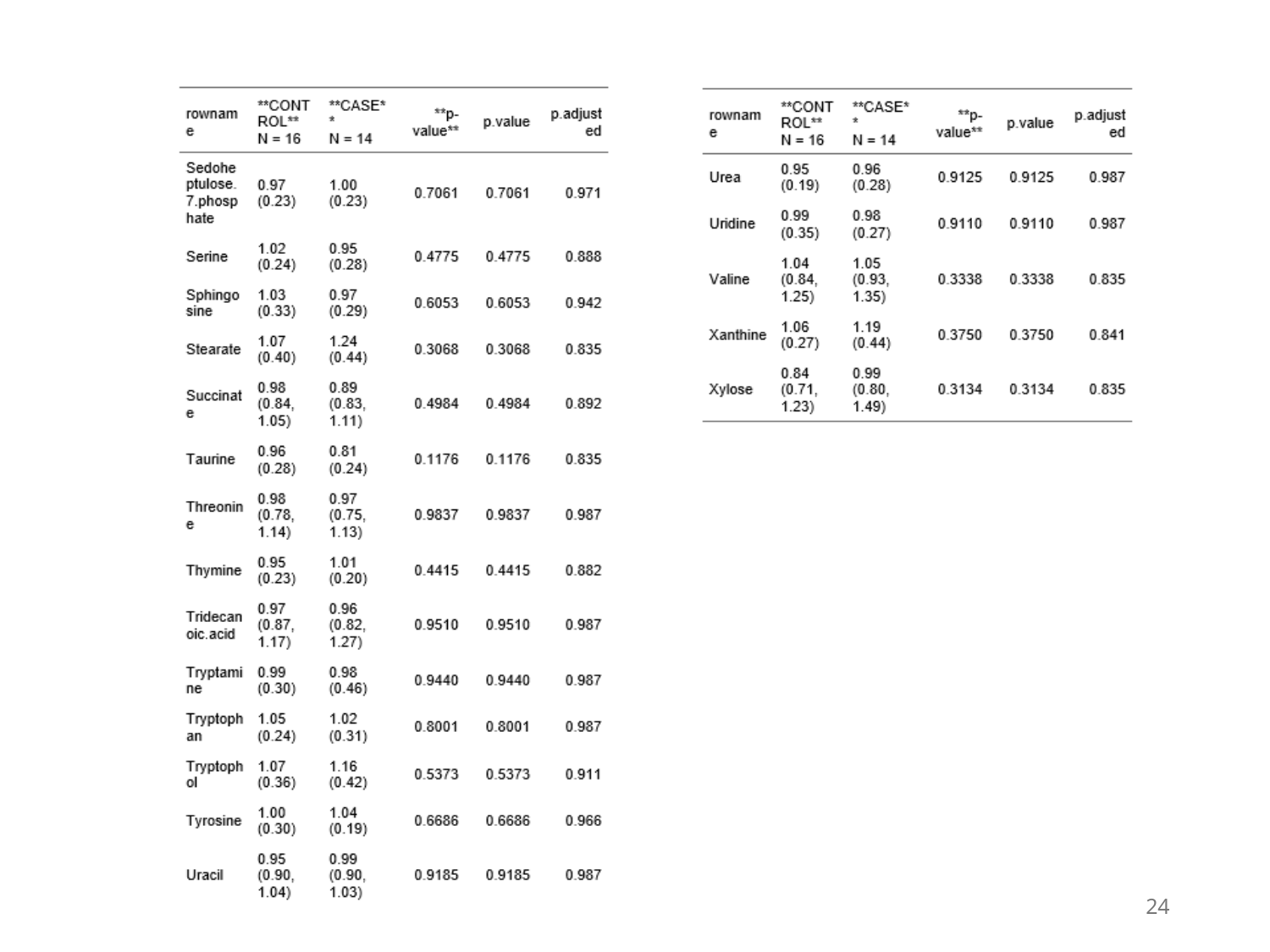

24
